# Modeling the Excess Cell Surface Stored in a Complex Morphology of Bleb-Like Protrusions

**DOI:** 10.1371/journal.pcbi.1004841

**Published:** 2016-03-25

**Authors:** Maryna Kapustina, Denis Tsygankov, Jia Zhao, Timothy Wessler, Xiaofeng Yang, Alex Chen, Nathan Roach, Timothy C. Elston, Qi Wang, Ken Jacobson, M. Gregory Forest

**Affiliations:** 1 Department of Cell Biology and Physiology, University of North Carolina School of Medicine, Chapel Hill, North Carolina, United States of America; 2 Wallace H. Coulter Department of Biomedical Engineering at Georgia Institute of Technology and Emory University, Atlanta, Georgia, United States of America; 3 Department of Pharmacology, University of North Carolina School of Medicine, Chapel Hill, North Carolina, United States of America; 4 Department of Mathematics, University of South Carolina, Columbia, South Carolina, United States of America; 5 Department of Mathematics, University of North Carolina at Chapel Hill, Chapel Hill, North Carolina, United States of America; 6 Lineberger Comprehensive Cancer Center, University of North Carolina School of Medicine, Chapel Hill, North Carolina, United States of America; 7 Departments of Applied Physical Sciences and Biomedical Engineering, University of North Carolina at Chapel Hill, Chapel Hill, North Carolina, United States of America; Irvine, UNITED STATES

## Abstract

Cells transition from spread to rounded morphologies in diverse physiological contexts including mitosis and mesenchymal-to-amoeboid transitions. When these drastic shape changes occur rapidly, cell volume and surface area are approximately conserved. Consequently, the rounded cells are suddenly presented with a several-fold excess of cell surface whose area far exceeds that of a smooth sphere enclosing the cell volume. This excess is stored in a population of bleb-like protrusions (BLiPs), whose size distribution is shown by electron micrographs to be skewed. We introduce three complementary models of rounded cell morphologies with a prescribed excess surface area. A 2D Hamiltonian model provides a mechanistic description of how discrete attachment points between the cell surface and cortex together with surface bending energy can generate a morphology that satisfies a prescribed excess area and BLiP number density. A 3D random seed-and-growth model simulates efficient packing of BLiPs over a primary rounded shape, demonstrating a pathway for skewed BLiP size distributions that recapitulate 3D morphologies. Finally, a phase field model (2D and 3D) posits energy-based constitutive laws for the cell membrane, nematic F-actin cortex, interior cytosol, and external aqueous medium. The cell surface is equipped with a spontaneous curvature function, a proxy for the cell surface-cortex couple, that is *a priori* unknown, which the model “learns” from the thin section transmission electron micrograph image (2D) or the “seed and growth” model image (3D). Converged phase field simulations predict self-consistent amplitudes and spatial localization of pressure and stress throughout the cell for any posited *stationary* morphology target and cell compartment constitutive properties. The models form a general framework for future studies of cell morphological dynamics in a variety of biological contexts.

## Author Summary

Individual cells must have the capability for rapid morphological transformations under various physiological conditions. One of the most drastic shape transformations occurs during the transition from spread to rounded morphologies. When this transition occurs rapidly, there is insufficient time for significant changes in surface area to occur, although the final size of the rounded cell indicates a significant reduction in apparent cell surface area at light microscope resolution. By contrast, high-resolution scanning electron micrographs of rapidly rounded cells reveal that a large amount of surface area is stored in a highly convoluted surface morphology consisting of bleb-like protrusions (BLiPs) and other small structures that are unrecognizable at lower resolution. This surface reserve is an important part of the mechanism that allows rapid and efficient large scale transformations of cell shape. Remarkably, although this convoluted morphology has been observed for decades, there has been very little effort recognizing and including this surface surplus in mathematical modeling of cell morphology and physiology. In this paper, we develop three complementary models to fill this void and lay the foundation for future investigations of the mechanisms that drive cellular morphological dynamics.

## Introduction

Cells maintain their structural integrity while being flexible enough to adopt a variety of shapes. In general, it is the cytoskeleton of eukaryotic cells that drives shape transformation leading to cell movement and provides the structural support to the cytoplasm and the means to resist external forces. The periphery of cells, consisting of the plasma membrane (PM) and the acto-myosin cortex, is highly dynamic to accommodate shape change. The plasma membrane (PM) consists of a high density of proteins [1] embedded in a phospholipid bilayer of 5–10 nm thickness, with a very limited ability to extend without rupture [2,3] but highly amenable to bending [4,5,6]. The thin (50–500 nm) layer of cytoskeleton structure immediately subjacent to the plasma membrane, known as the cell cortex, consists of a dense F-actin network that is cross-linked by actin binding proteins and is amenable to contractility mediated by myosin motors. Interposed between the cortex and the PM is a thin spectrin-actin network, forming a ‘fishnet’ with a mesh size of ~100 nm [7,8]. This structure is anchored both to the PM and cortex by adaptor proteins. In the following, we term the plasma membrane and spectrin-actin network as the “cell surface”.

Previously we [9] suggested that most dynamical shape changes exhibited by non-spread (rounded) cells originate from a membrane-cortex folding-unfolding process and an excess of cell surface area is a necessary requirement for such changes. We investigated the dynamics of periodically protruding cells and hypothesized that the plasma membrane and thin cortical layer remain coupled during all stages of shape transformation. We also assumed that densely compressed cell surface folds and small protrusions could be kept intact by the underlying actin-myosin network residing in the cortex proper. While this notion may be applicable to many shape transitions occurring in non-spread cells, in this paper we reconsider this hypothesis in context of one of the most drastic changes of cell shape: the transformation from a fully spread to a rounded state.

If a cell transitions from a spread to rounded state while maintaining a constant volume, it will experience an excess of surface area over the minimum needed to cover the enclosed volume. Because this process typically happens rapidly (~30s-), there is insufficient time for excess membrane to be internalized by endocytosis. Thus, another mechanism for storing surface area at the plasma membrane must exist. Indeed, there is significant evidence from both electron and fluorescence microscopy that during the rounding process the cell surface adopts a tightly folded morphology [9,10,11,12].

While there are a number of models for cell shape, most of them treat the cell surface as smooth [13,14,15,16,17] and do not take into account the possibility that rounded cells store excess surface area in a dense distribution of bleb-like protrusions (BLiPs). Thus, new modeling approaches are needed to understand the dynamics of cell shape changes that involve active use of this surface storage. We introduce three complementary modeling approaches, each incorporating the concept of excess surface area. The first approach is a 2D model based on a thin cell surface structure that is coupled to a thicker, contractile actomyosin layer. This model allows us to investigate the folding of the excess surface and to estimate the bending energy in different configurations. The second approach is a random “seed and growth” model that produces 3D morphologies consistent with the distributions of BLiP size and number estimated from scanning electron micrographs. This model yields insight into how large numbers of BLiPs are efficiently packed on the cell periphery.

The third approach is a multi-compartment phase field model. By faithfully capturing the physical properties of the cortex, cytosol, and cell surface, the model predicts the stress and pressure distributions associated with a highly folded 2D morphology and a dense distribution of 3D BLIPs. Phase field models have been widely used to study complex systems comprised of distinct material phases and their adjacent interfaces. When the separate material phases are immiscible, the phase field approach is to prescribe a finite thickness of a “diffuse” interface within which there is a mixture of the two materials [18,19]. The phase field method is an alternative to sharp interface methods; in both methods the shape and evolution of the sharp versus diffuse interface are part of the solution. For every pair of adjacent material components, a phase field variable is introduced that interpolates from one material phase to the other through the finite thickness boundary. Phase field models have been employed to describe shapes of lipid bilayer vesicles in which the surface tension and Helfrich bending energy are approximated using a bulk energy defined within the diffuse interfacial layer [19]. Phase field models have been applied to many interfacial problems including liquid drops, multiphase complex fluids [20], and fractures in solid-state materials [21].

The phase field model simulations achieve separate goals. From either a 2D transmission electron micrograph or a 3D image reconstruction of the cell morphology, the model “learns” the spontaneous curvature functional of the rounded, BLiP-rich, morphology. Since the phase field model faithfully captures material properties of each cellular compartment, the model converges to the cell target morphology while constructing self-consistent stress and isotropic pressure distributions for the cell surface, cortex and cytoplasm, as well as estimating the nematic orientation within the cortex.

Storage of excess cell surface in folds or bleb-like protrusions at the periphery is likely to be important for a variety of rapid cell shape changes, taking place over a time scale of a few minutes or less, such as those that occur in forms of amoeboid migration or either within or in the transitions between the phases of mitosis. It seems likely that rapid cell shape changes can be accomplished more quickly by calling upon a reserve of excess membrane stored in the BLiP distributions rather than relying on extensive membrane-cortex remodeling and exocytosis. Thus, the theoretical approaches presented here should be applicable in a number of different biological contexts.

## Results

### Cell rounding produces an accumulation of excess surface at the cell periphery

**Fig 1:**
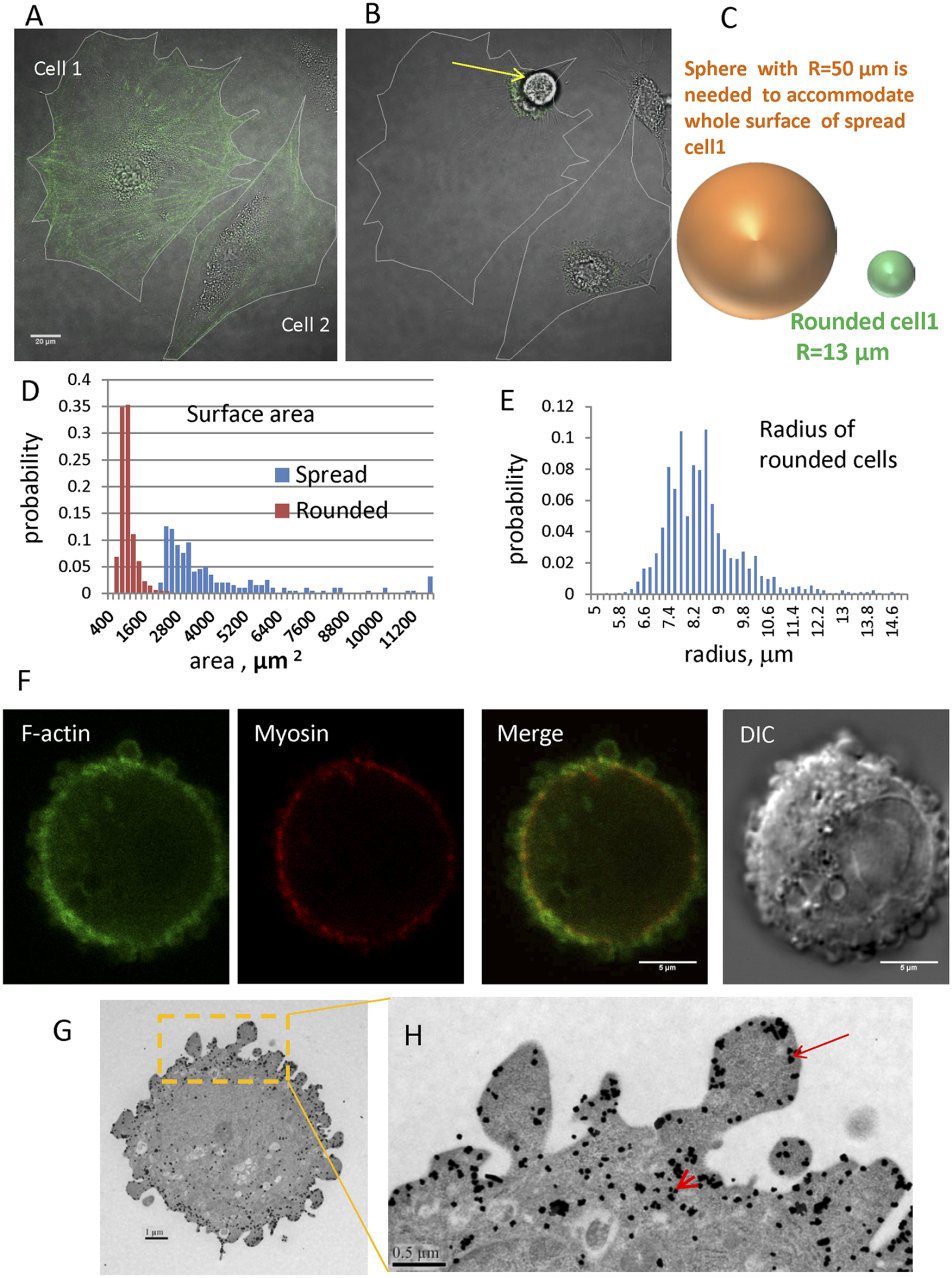
Transition from spread to rounded states and localization of F-actin and myosin in the cortex of a typical rounded cell and cortical structure. A. Merged DIC and fluorescence image of spread Swiss 3T3 fibroblasts stably transfected with Lifeact-RFP (green) cells with clearly visible signal from stress fibers. Cell outlines traced from image. Bar = 20μm. B. Image of the same cell after trypsin-induced detachment. The white outline shows the former spread shape. Yellow arrow points to the rounded cell. C. Cartoon compares the radius of a sphere required to accommodate the cell volume from the spread state (R = 50μm) versus the rounded state (R = 13 μm) for the cell on the left. D. Distribution of spread and rounded cell areas. E. Distribution of rounded cell radii. F. DIC and confocal fluorescence images of rounded CHO cell stably expressing GFP-Lifeact (green) and RFP-MLC (red) Bar = 5 μm. G. Transmission electron microscopy image of GFP immunogold staining of rounded CHO cells with stable expression of Lifeact-GFP. Black dots which represent gold particles show the position of actin filaments. Bar = 1 μm. H. Inset shows outlined region in G at higher magnification. Arrow points to F-actin immediately underlying the plasma membrane in BLiPs: arrowhead points to the F-actin in the cortex closer toward center of the cell. Bar = 0.5 μm.

When spread cells (Fig 1A) are chemically detached from an underlying substrate, they rapidly transition to a rounded state on a characteristic time scale of ~30-60s (Fig 1B). Numerous studies suggest that in media with constant osmolarity, cell volume is stable [3,22]. We estimated cell volume by reconstructing 3D geometries from Z-stacks of spinning disc fluorescence images of cells undergoing rounding. The mean volume for Chinese hamster ovary (CHO) cells in the spread state is 6.5±2.82*10^3^ μm^3^, while the mean volume in the rounded state is 5.7±2.30*10^3^ μm^3^, indicating a slight decrease in cell volume after rounding. Because this slight decrease in cell volume would only increase excess surface area, in all our models, we assume that cell volume remains constant during rounding.

The surface area of a spread cell is estimated as twice the area measured from images to account for dorsal and ventral surfaces. In reality cells are not completely flat and have more surface area due to finite thickness, particularly around the nucleus. Therefore, we are underestimating the surface area of a spread cell. In the rounded state, the minimal surface area needed to enclose the measured cell volume can be found by assuming the cell is spherical and calculating the radius. For example, the cell 1 in Fig 1A has a surface area of ~ 31000 μm^2^ while the surface area needed to enclose the rounded state is only ~ 2200 μm^2^. Therefore after rounding, this cell has ~ 14 times more surface area than is required to enclose its volume. This image presents an extreme case of surface area excess. For cell 2 in Fig 1A, which is less spread before detachment, an excess surface area of about five times the required amount is accumulated following rounding. It is important to note that the amount of excess surface area that is accumulated during rounding depends on cell type and characteristics of the spreading and detachment for individual cells. Using DIC and fluorescence microscopy, we studied populations of cells before and after detachment, and individual cells rounding during trypsinization. The histogram in Fig 1C presents the distribution of surface areas for spread CHO cells (blue bars; population mean = 4310±3600 μm^2^, N = 199) and cells immediately after rounding (red bars; population mean = 892±284 μm^2^, N = 1646). The distribution of rounded cell sizes is narrow with majority of cell radii (Fig 1E) being between 7 and 9.5 μm (mean = 8.36±1.24 μm; N = 1646). Separate experiments, where we followed the change in morphology of individual cells during rounding, demonstrated that for CHO cells the average excess surface, defined as the ratio of spread cell area to that required to smoothly cover a sphere with radius corresponding to that of the rounded cell, accumulated due to detachment and rounding is 3.8± 2.06 with maximum value of 12 (N = 99).

To gain insight into how much excess surface area can be stored in BLiPs, we first consider the case of a rounded cell uniformly covered with equally sized sphererical BLiPs.

It is easy to show (S1 Appendix) that as spherical BLiPs become smaller, more excess surface area can be accommodated. The maximum possible surface excess that can be stored in the equally sized sphererical BLiPs is 5 (in the limit of BliP radius *r*→0). The fact that we observed rounded cells with the surface excess as high as 14, means that cells utilize a more efficient packing strategy. Also no limits on the surface excess ratio would be imposed if we did not require BLiPs to be spherical, but rather allow for an arbitrarily high curvature of the surface, as occurs, for example, in tubules. Yet, the majority of BLiPs appear to be rounded immediately after detachment. These considerations suggest that the actual morphology of the folded cell surface is dictated by a balance between the necessity to pack tightly a very large number of BLiPs and the necessity to generate, regulate, and maintain significant surface curvature.

### Cortex architecture informs the 2D discrete geometric model

To better understand the process of packing cell surface excess into a convoluted surface morphology, we constructed a 2D geometric model designed to produce BLiPs. We hypothesize that the cell surface and underlying contractile cortex form a two-layer structure that is coupled at certain fixed points. The first layer, which we term the cell surface, is passive and consists of the plasma membrane and membrane associated cytoskeleton. This layer is assumed to be similar to the spectrin-actin network that is coupled to the plasma membrane of red cells [23]. Such structures have been shown by Kusumi and co-workers to exist in many other cell types [8]. The membrane associated cytoskeleton has been termed the membrane skeleton fence [8]. It is basically a very thin filamentous meshwork that provides a “fishnet” with a mesh size of approximately 100 nm immediately underlying the PM. This layer is coupled to the plasma membrane via adaptor proteins including the ankyrin and ERM families as well as by interactions of the membrane skeleton fence with lipids in the inner monolayer of the PM. The layer is thought to be passive undergoing only thermal motions, serving to anchor some transmembrane proteins and restrict the free diffusion of others. We assume that this thin layer is coupled via adaptor proteins to a thicker, active contractile layer containing actin and myosin. This view of the cortex-cell surface couple is consistent with that advanced by Charras et al (2006) [24] in the context of spontaneously blebbing cells.

Additional evidence for this structure comes by imaging employing confocal and electron microscopy. Fig 1F shows a confocal image of the actin-myosin cortex in rounded cells as visualized with GFP-lifeact and RFP-myosin merged with a DIC image of the same cell. The green signal for Lifeact marks F-actin filaments associated with the folded morphology of the cell periphery and this fluorescent signal originates from both the thin layer immediately subjacent to the membrane and the thicker contractile layer. The fluorescent signal from myosin (red) shows that this protein is localized mainly to a thin circle located below the BLiPs and more toward the cell interior. S1 Fig presents Z-stack images of the same cell. From this image, it is clearly visible that the convoluted morphology covers the whole cell. Fig 1G and 1H shows immunogold TEM images of GFP-lifeact where F-actin is seen underlying BLiPs (arrow) and also in a layer closer toward the center of the cell (arrowhead).

**Fig 2:**
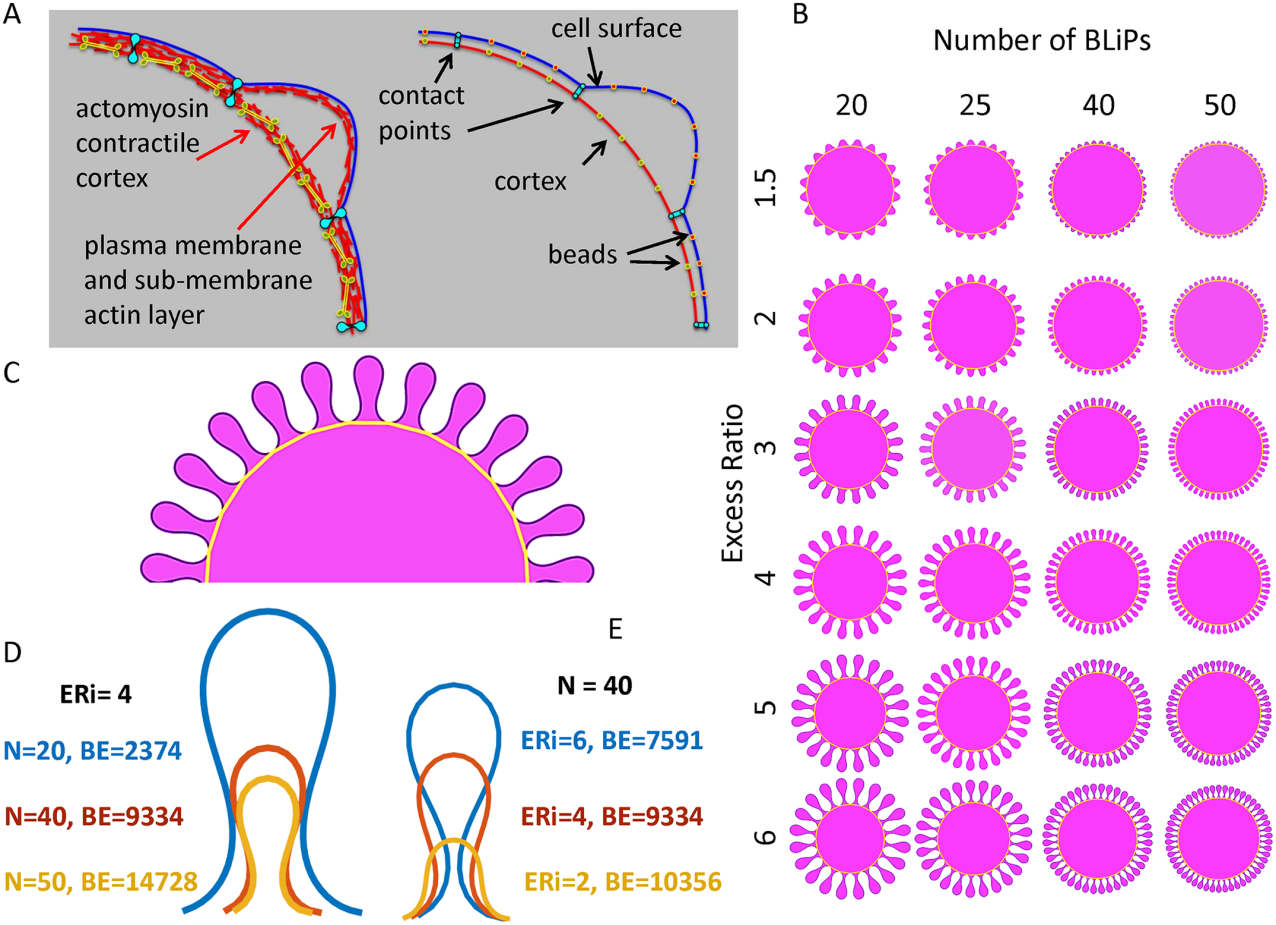
The discrete geometric model. A. Two layer cortex model (left) and its bead-spring representation with notations employed (right). B. Folded geometries as a function of the fold number and the excess surface ratio. C. A portion of a model cell with BLiPs at steady state where the gold line represents the cortex. D. The shape of three single folds extracted from simulations for initial excess ratio, ER = 4 with different numbers of folds, N: 20 (blue), 40(red) and 50(yellow). E. The shape of three single folds extracted from simulations for number of folds N = 40 with different initial excess ratio, ER,: 6 (blue), 4 (red), 2 (yellow). All calculated shapes were scaled to have the same unit area inside the outer perimeter. The bending energy (BE) is presented for each configuration.

To construct the 2D geometric model introduced qualitatively above, we implement a two-layer architecture in a 2D bead-spring model of the cell membrane and cortex (model description in Methods). The bead-spring model consists of two-layers (Fig 2A and S2 Fig), where one layer (outer layer) represents the membrane and underlying actin mesh (i.e. the cell surface) and the other layer (inner layer) represents the actomyosin-rich contractile cortex. In each layer, beads are connected pairwise by springs and contact points serve to connect the two layers. By minimizing the bending energy we explored the steady-state shapes of BLiPs generated during cell rounding when the cell is rapidly presented with a substantial excess surface. For simplicity we define excess surface ratio (ER) as a ratio of perimeters of the surface layer and contracted cortex. Here we define a normalized total bending energy (E) as for

$$E=\oint_{s}\kappa^{2}ds\approx \frac{L}{N}\sum_{i=1}^{N}\kappa_{i}^{2}$$ (1)

where κ is the local curvature measured between two neighboring beads, *L* is the perimeter, and N is the number of beads in the outer layer [25,26,27,28,29].

The number of contact points determines the number of folds (M). In the simulation the total Hamiltonian of the two-layer system is minimized with the result that a folded configuration of outer layer is produced. Fig 2B shows the resulting shapes as a function of both *M* and ER. Fig 2C shows a portion of a model cell with BLiPs at steady state where the gold line represents the contractile part of the cortex with contact points. While the appearance of folds is expected, the shape of folds and the bending energy stored in each configuration is of particular interest.

Fig 2D gives a comparison of the fold configuration for several different sets of parameters with the calculated bending energy for each shape. Inspection of the fold shapes shows that in order to accommodate more surface, the folds tend to develop long necks. (Note that in case where there is heterogeneity in the size of folds, this effect would allow small folds to grow under larger ones, an effect that permits accommodation of more excess surface.) The smallest possible bending energy will be achieved when ER = 1 and M = 0 (no surface excess and no BLiPs). For a given value of ER, the energy increases with the number of BLiPs (Fig 2D and S2 Fig). However, the bending energy is decreasing while the excess surface ratio is increasing. Although this result looks counterintuitive, it can be explained. The local curvature is the inverse of local radius. Folds with a longer perimeter have bigger inner radii which substantially decreases bending energy with the square of local radius (Fig 2D and S3 Fig). Thus, morphologies with longer perimeters corresponding to larger ERs will have lower energy compared to the shapes with the same number of folds but with shorter perimeters (i.e. smaller ERs). The analysis of the area which is stored inside the folds (i.e., volume in 3D) shows that for the same surface excess, more area is stored in folds when the number of folds used for accommodation of this surface surplus is smaller (S3D Fig).

### BLiP size measurements

**Fig 3:**
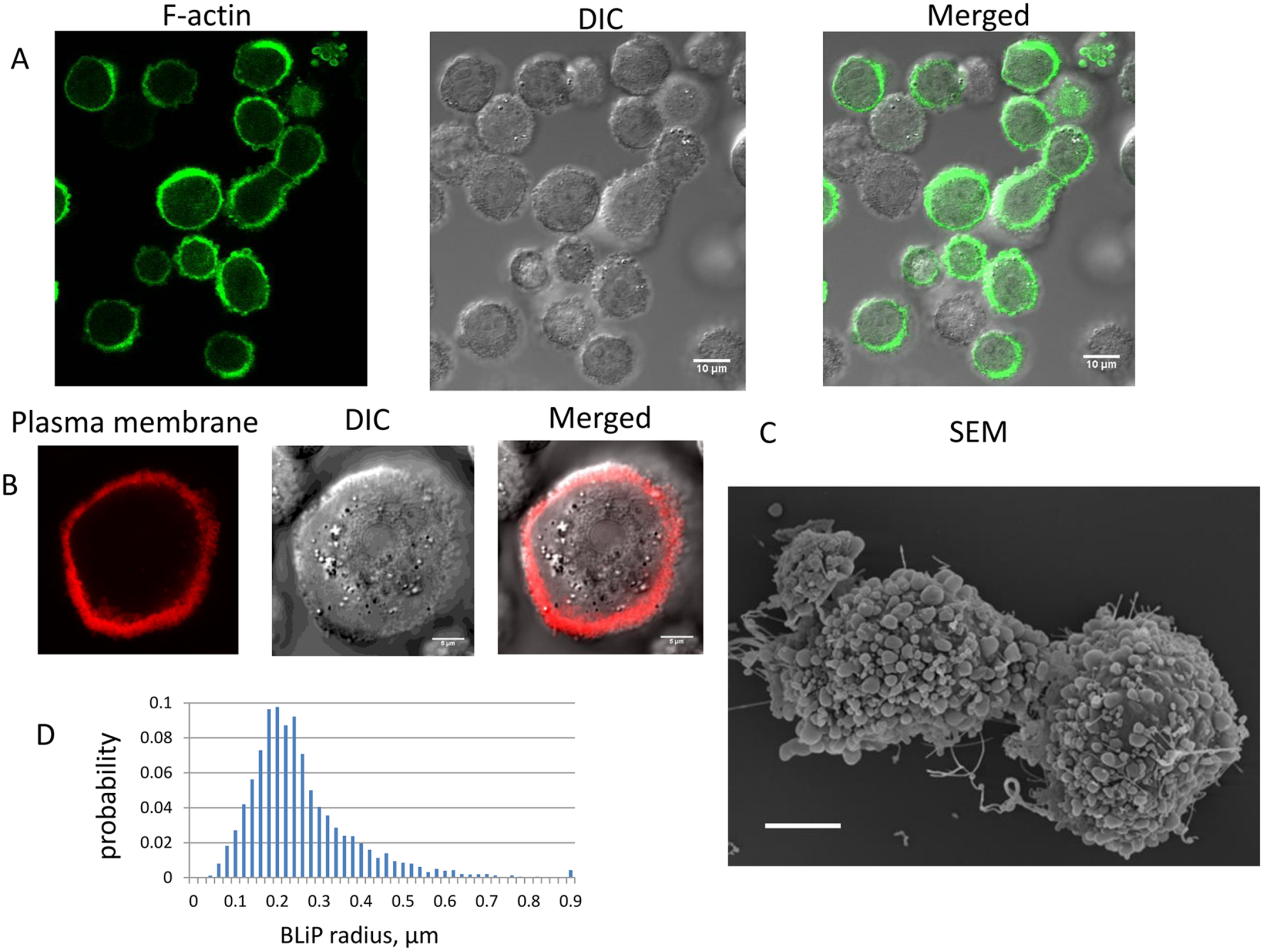
Morphology of rounded cells. A. Fluorescence, DIC and merged images of rounded CHO cells stably transfected with Lifeact-GFP. B. Fluorescence, DIC and merged images taken near the equatorial plane of a rounded CHO cell with the fluorescence signal coming from the PH domain of PLC-delta fused to EGFP that marks the inner leaflet of the plasma membrane (Bar = 5 μm). C. Scanning electron microscope image of the rounded state (Bar = 5 μm). D. BLiP radii distribution for N = 7096 BLiPs.

At the resolution achievable by standard fluorescence microscopy, the convoluted cell surface often appears as a thickening of membrane and cortical stains (Fig 3A and 3B). However, surface morphology can be imaged at higher resolution using both scanning (SEM) and thin-section transmission (TEM) electron micrographs (Figs 3C and 1H, respectively). At this scale, bleb-like protrusions (BLiPs) and other cell surface protuberances are clearly visible. Note that only fully spread cells have a smooth surface essentially devoid of protrusions (S4 Fig).

To determine length, area and volume metrics of BLiPs, we manually segmented SEM images of cells that were fixed after rounding (S5 Fig). Each protrusion was approximated as a sphere and the area of the protrusion visible on the image was interpreted as a two dimensional projection of that sphere. We calculated the radius that corresponds to a projection of that size, and consider it as the radius of the BLiP. The distributions of BLiP radii derived from 10 SEM images (25 cells) that include 7096 BLiPs is presented in Fig 3D. We find that the distribution of radii is skewed with a preponderance of small BLiPs and a decreasing frequency of larger BLiPs. The mean BLiP radius is R = 0.25 μm with a median of 0.22 μm and mode of 0.19 μm.

It is important to mention that during the processes of detachment and rounding some part of the cell surface can be lost due to incomplete detachment from the substrate or because it remains in retraction fibers. However, the area remaining in retraction fibers is quite small. Using SEM images from cells that spread for 24 h and rounded 5 minutes before fixation, we estimated that the surface area that might be stored in retraction fibers represents between ~0.5–5% of the cell surface area in the spread state.

### BLiP packing on the cell periphery

**Fig 4:**
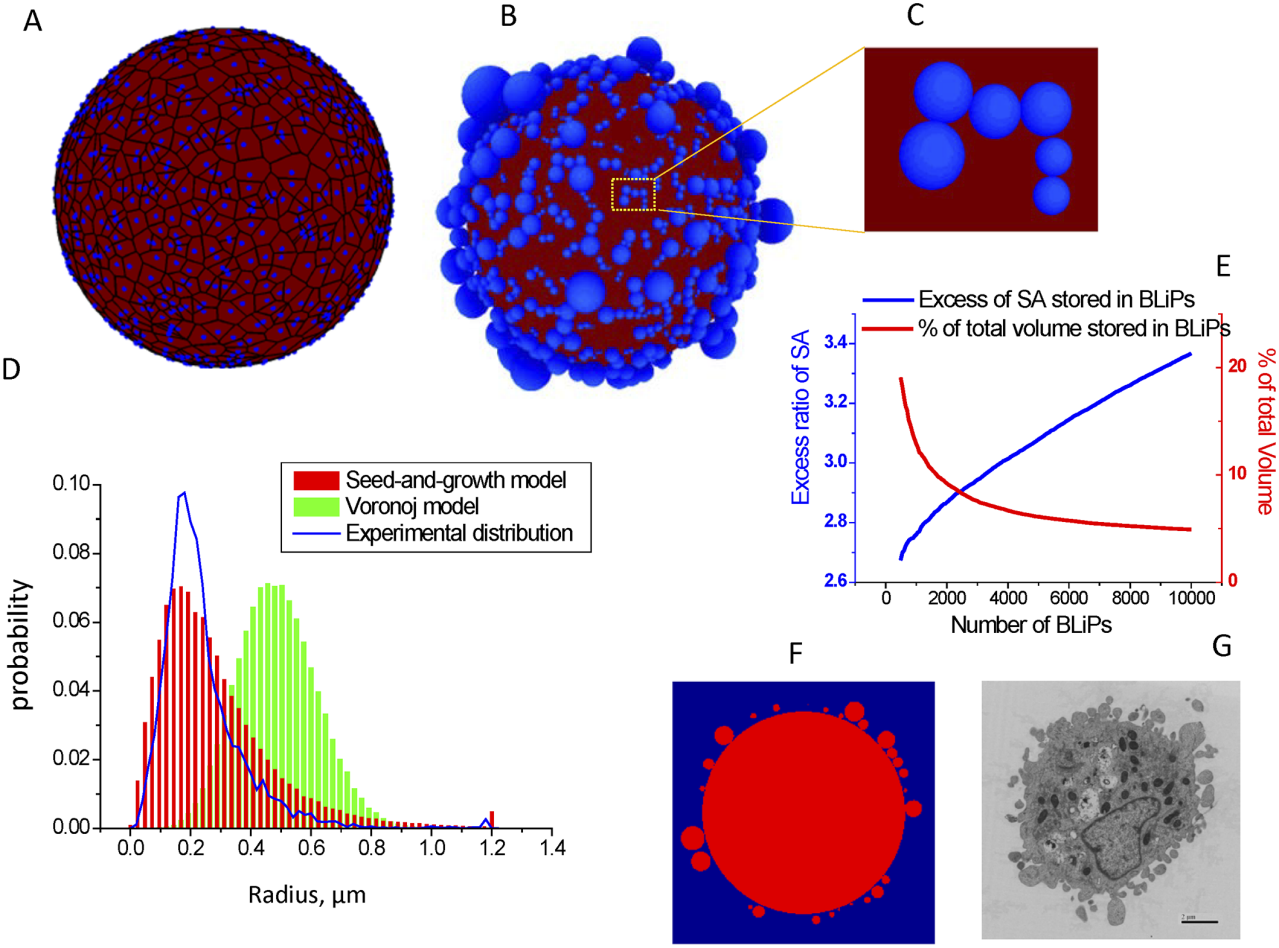
BLiPs morphology generated by seed-and-growth models. A. A realization of the Voronoi diagram. The seeds and boundaries of Voronoi cells are shown in blue; the Voronoi polygons are shown in red. B. A realization of the “seed and growth” model. C. Magnified view of rectangle in (B). D. Distribution of BLiP sizes: Voronoi model (green bars) and “seed and growth” model (red bars) normalized using the assumption that average rounded cell radius is 8 um; the blue line shows the experimentally obtained distribution. E. For the “seed and growth” model, plots of % surface area stored in BLiPs and % of total volume stored in BLiPs as a function of the number of BLiPs. F. A cross-section of “seed and growth” model. G. Transmission electron micrograph of a section of a rounded cell (Bar = 2 μm) for comparison to F.

To investigate how the large number of BLiPs required to accommodate the excess cell surface are packed on the cell periphery, we constructed two models. As a plausible starting point, we employed a Voronoi approach, in which a spherical ball of radius $R=\sqrt{S/4\pi }$ contains the surface area, S of the spread cell before detachment and rounding; *n* seed point locations are randomly sampled from a uniform spatial distribution on the ball. The ball is then partitioned by a Voronoi tessellation according to the *n* seed points (Fig 4A) such that any point in each Voronoi cell is closer to the parent seed point than any other seed point. The area of each Voronoi cell is then determined. In this configuration, we assumed that, upon the cell rounding to its final state with the “BLiPed” morphology, each Voronoi cell of area *v* morphs into a spherical BLiP with radius $r=\sqrt{\nu /4\pi }$. Fig 4D demonstrates that, constructed in this way, the distribution of BLiP radii has a well-defined length scale with the bell-shape distribution, which is not consistent with the skewed distribution of experimental data. This result arises from the fact that Voronoi cells corresponding to two very closely positioned seeds are not necessarily small themselves, as might be expected from two closely positioned BLiPs.

In order to mitigate this effect, we introduce an alternative 3D “seed and growth” model (Fig 4B and 4C), in which BLiP radii are proportional to spacing between randomly distributed seeds. In this model, spheres are generated from each seed point by increasing their radii at a uniform rate. Simultaneously, the locations of the seed points are moved outward radially at the same rate, so that the spheres always remain tangent to the cell. When one sphere encounters another, it stops growing. When all spheres have stopped growth, a spherical cell coated by different sized BLiPs is produced (Fig 4B). The resulting BLiP radius distribution in Fig 4D is more consistent with the experimental distribution than that produced by the Voronoi model. The generated structure is also consistent with SEM image data, which show approximately spherical BLiPs largely covering the cell but with some areas devoid of BLiPs. We reproduced 2D cross-sectional views from the simulated 3D geometries (Fig 4F); these show similarity to the thin section TEM images of rounded cells (Fig 4G) where some of the BLiPs appear to be detached from the cell body because BLiPs are not always sectioned through their centers.

In the “seed and growth” model, a larger number of BLiPs results in a higher surface area excess ratio (Fig 4E) and a smaller percentage of the cell volume stored within the BLiPs, which is consistent with our simplified estimations based on an 2D equal-sized BLiP distribution. In principle, BLiPs that are not of equal size could allow a more efficient packing of excessive cell surface (with smaller BLiPs filling the space between larger ones), which is important for accommodating very high excess ratios (>5). However, in this model the packing is still inefficient because it always generates areas devoid of BLiPs. A potential improvement to our model might be to incorporate stochastic seeding of new BLiPs and occasional “shrinking” BLiPs that are in contact, so that BLiPs are dynamic and continue to adjust themselves toward the most efficient filling of the available space. Such a “seed, growth, and shrinking” model would be consistent with the BLiP dynamics observed in our experiments, but is beyond the scope of the current paper. Another potential improvement would be to make final BLiP size proportional to the rate of expansion of the BLiP; this has been found to be the case in an earlier study of blebbing cells [30].

### A phase field model can learn the spontaneous curvature of the cell surface from 2D transmission electron micrographs or 3D image reconstructions

**Fig 5:**
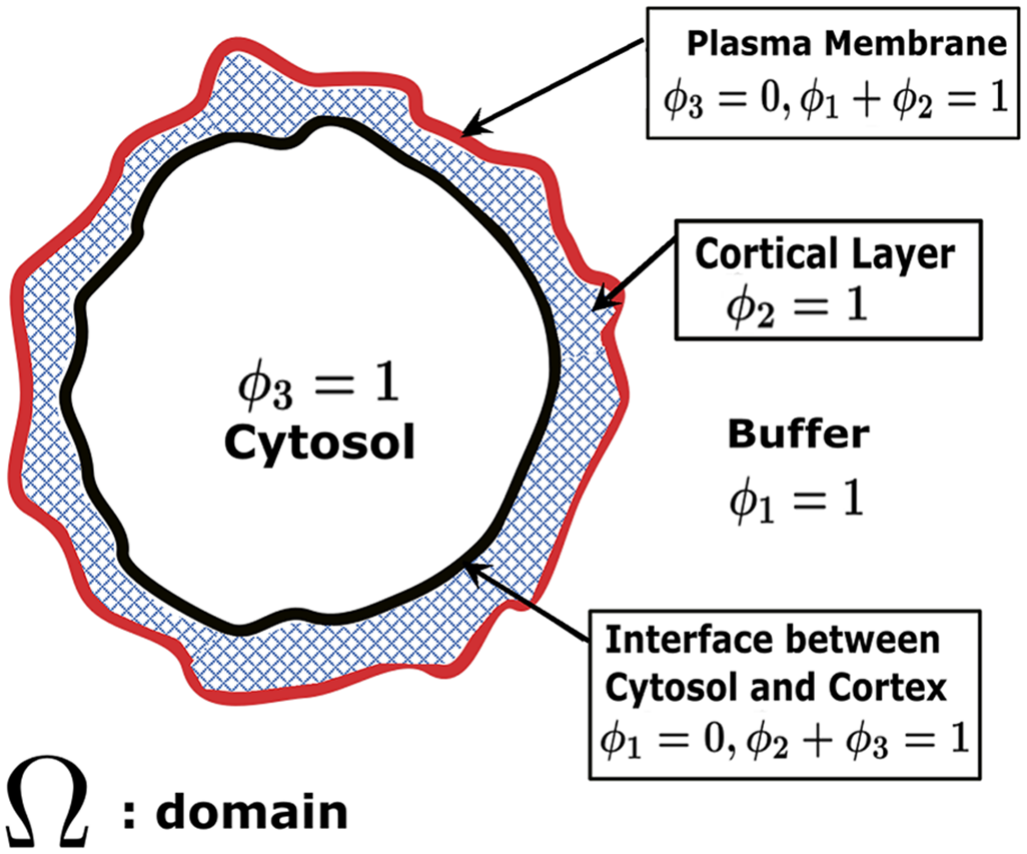
Schematic for the phase field formulation of a cell in an aqueous medium. *ϕ*_*1*_, *ϕ*_*2*_, *ϕ*_*3*_, represent volume fractions of the external aqueous medium, the nematic cortex as schematically depicted by the cross-hatched region and the cytosol, respectively, with *ϕ*_*1*_+*ϕ*_*2*_+*ϕ*_*3*_ = 1. The entire computational domain is denoted as Ω. The cell surface is defined by the level sets *ϕ*_*1*_ = *ϕ*_*2*_ = 0.5, while the cortex-cytosol interface is defined by *ϕ*_*2*_ = *ϕ*_*3*_ = 0.5. Note that we penalize coexistence of three phases.

The preceding models help build mechanistic intuition, yet, while predictive, they do not capture all of the essential physics of the rounded phenotype. In order to approach this goal, we formulated 2D and 3D phase field models for a cell immersed in the aqueous extracellular environment. The model is formulated in 3 space dimensions (3D), but it also restricts to 2D for purposes of modeling a cell cross-section. In our case, we have three phases (exterior aqueous medium, cortex, interior cytosol) and two diffuse interfaces. The external aqueous medium and interior cytosol are modeled as viscous fluids with specified viscosities and the cortex is modeled as a nematic (liquid crystal) gel [31]. The first diffuse (i.e., finite thickness) interface is what we have termed the cell surface, consisting of the plasma membrane and very thin underlying filamentous “fishnet”, that separates the aqueous medium and the cortex proper. As described below in Methods, a particular level set function in the phase field formulation will afford our definition of the “cell surface”. The second diffuse interface is the cortex-cytosol transition layer. Fig 5 is a 2D schematic of a 3D cell cross-section with individual components and diffuse interfaces labeled, along with the phase variables defined below. (We do not explicitly model the nucleus within the cytoplasm for this paper since we are primarily concerned with the stationary rounded morphology.) A more complete mechanical formulation giving the total system free energy in terms of its components is found in Methods.

### Phase field modeling of a 2D cell surface morphology with excess “perimeter”

**Fig 6:**
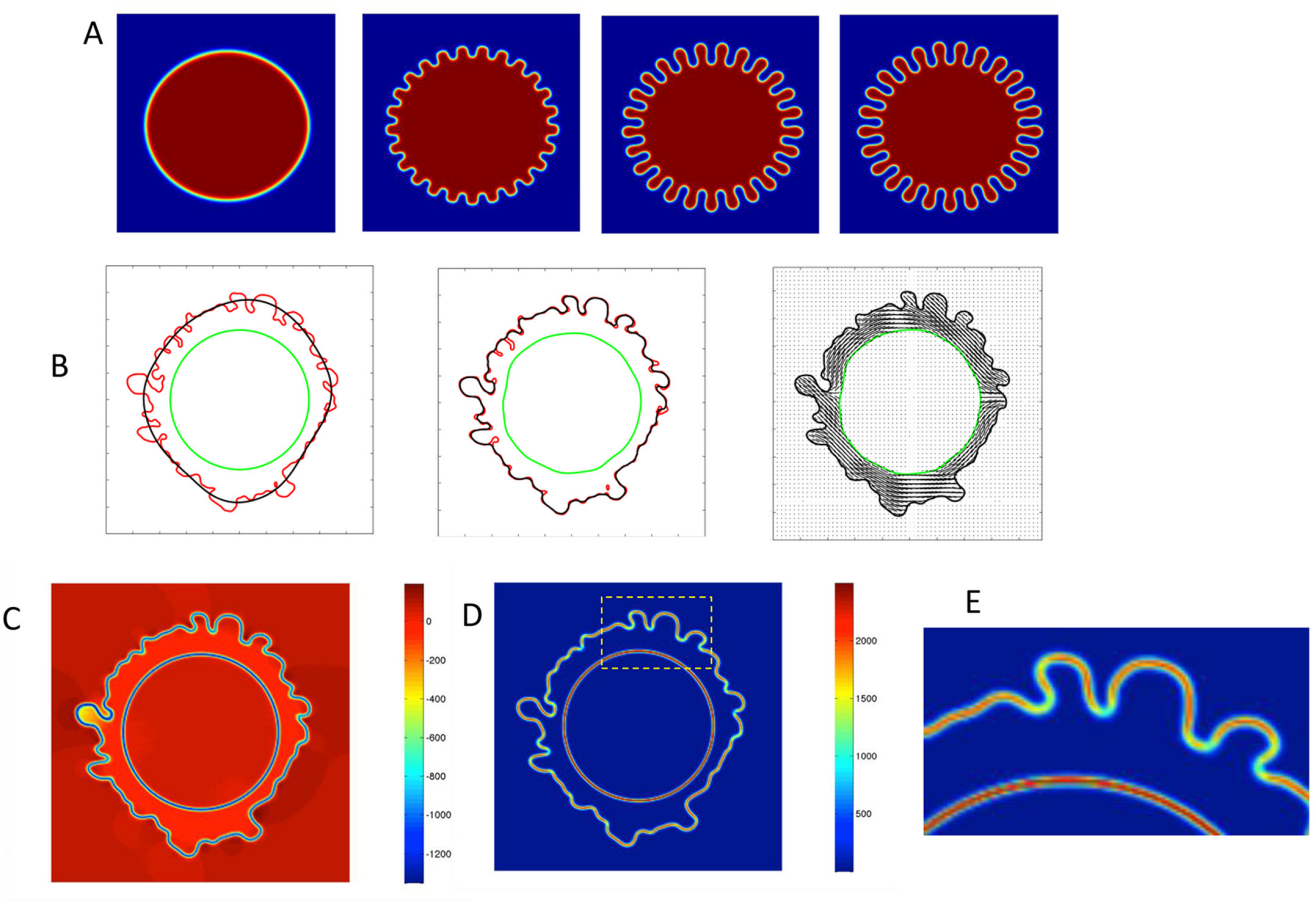
Application of the phase field model to 2D target images. A. Proof of principle of the 2D phase field simulation. From left to right, convergence from circular initial data to a target cell surface morphology with 25 uniform, equally spaced, “BLiPs”. B. Convergence of a 2D phase field simulation to a 2D, TEM image of a representative cell surface morphology. The red curve in panels 1–3 is the cell surface obtained from a 2D TEM micrograph, which serves as the target of the phase field model. The black contours in each panel are the initial data (left panel) which evolves to the actual cell surface in the phase field simulation. The green contours depict the interface between the cortex and interior cytosol. In the right panel, the F-actin filament orientational distribution within the nematic cortex is superimposed, as predicted by the phase field model. In these simulations, the Flory order parameter, $\sqrt{h_{1}/h_{2}}$, is set to 1. C. Phase field predictions of the pressure distribution. D. the first invariant (trace) of the dominant stored stress, the Ericksen stress, for the converged stationary morphology shown in B, right panel. E. A blow-up of the trace of the Ericksen stress inside the dashed yellow rectangular domain in D. The color bars for C, D, and E are in units of Pascals.

We summarize the key numerical results of the phase field modeling of a 2D cell surface morphology due to an imposed excess arc length enclosing the 2D area. We require a 2D image of the membrane morphology, taken from 2D transmission electron micrographs. From the image file, we posit an initial smooth membrane boundary, and then evolve the phase field model while adjusting the spontaneous curvature function *C*1 until the model converges to the image dataset. We first illustrate the ability of the phase field model to match an arbitrary specified 2D boundary by “learning” the spontaneous curvature function; the results are shown in Fig 6A for an illustrative benchmark in which the cell perimeter contains 25 regularly spaced, uniform “BLiPs”. (In 2D, this is achieved by superimposing the appropriate Fourier mode on a circle.) Next, we used as input the actual periphery of a rounded cell from a 2D transmission electron micrograph (TEM) image. The results in Fig 6B show the convergence of the phase field membrane morphology to the TEM image, where the nematic phase ordering (representing F-actin orientation) in the cortex is depicted. This result assumes tangential anchoring condition of F-actin at both cortical diffuse interfaces.

Fig 6C–6E shows the phase field predictions of the pressure distribution (C) and the first invariant of the dominant stored stress, the Ericksen stress, for the converged stationary morphology (D,E) shown in Fig 6B. These results reveal the orders of magnitude as well as spatial localization of pressure and stored stress for the target 2D morphology.

### Phase field recapitulation of 3D BLiP morphologies and associated stress maps

**Fig 7:**
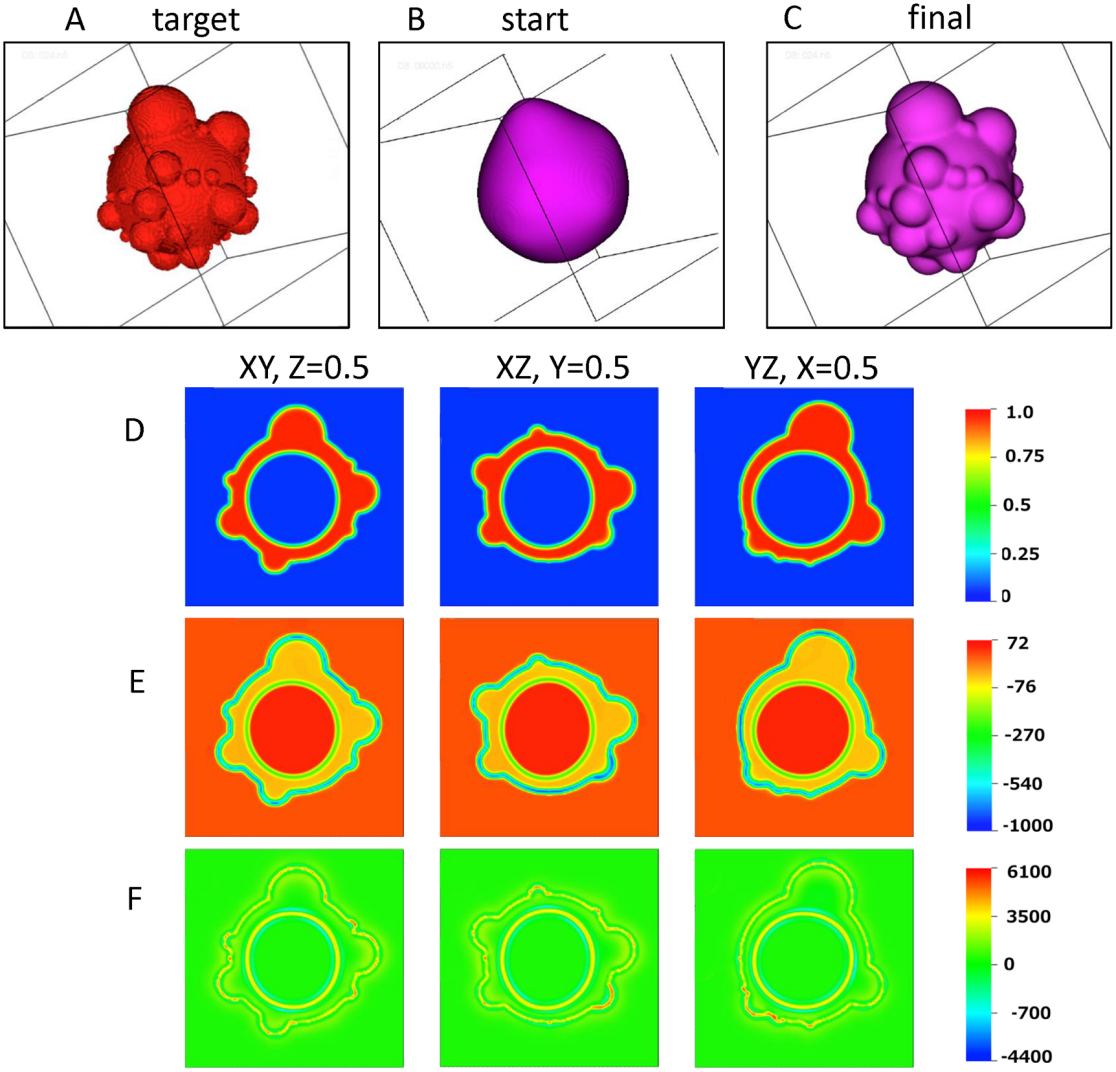
Phase field simulation showing convergence to a target 3D cell morphology image. (A) The target 3D cell morphology. (B) The assumed initial data for the cell surface. (C) The converged cell surface from the phase field simulation. (D) 2D planar slices at z = 0.5, y = 0.5 and x = 0.5, respectively, of the converged 3D phase field simulation shown in C. The color bar depicts level sets of the cortical phase variable *ϕ*_*2*_ to delineate the cortex (red is the level set *ϕ*_*2*_ = 1) from the pure external aqueous medium and pure interior cytosol (both blue since *ϕ*_*2*_ = 0), while both diffuse interfaces are depicted by the color interpolation between these level sets of *ϕ*_*2*_. (E) Corresponding pressure distributions (units of Pa) in the 2D slices shown in (D). (F) Distribution of the trace of the Ericksen stress (units of Pa) in the 2D slices shown in (D). The color bar for E, F is in units of Pa. For these calculations the Flory order parameter is set to 1.

A 3D simulation is depicted in Fig 7 to demonstrate the capability of our phase field model to converge to a *target* 3D cell surface morphology. The excess surface area ratio for this illustration is *s*_*0*_ = 3. Because it is impossible to reproduce a full 3D morphology of a rounded cell from a single scanning electron micrograph, we use the “seed and growth” model to simulate a 3D cell surface target. This model (Fig 4) provides a 3D surface morphology consistent with the measured BLiP size distribution data; therefore, we posit the output image from this model as the target morphology for the 3D phase field simulation. As shown earlier in 2D and here in Fig 7 in 3D, the phase field model converges to the target 3D morphology from an initial posited surface, while satisfying the volume and excess surface area constraints. The model does so by iterating the spontaneous curvature function until all constraints are satisfied; once converged, the model then yields the pressure and stresses within the cell surface and cortex that are self-consistent with the 3D surface morphology and constitutive properties of the exterior and cell compartments.

In Fig 7A, the target cell morphology is shown. The evolution of cell morphology, from an initial rounded cell guess to the target cell shape, is provided in (Fig 7B and 7C). Fig 7D depicts 2D projections in three mutually orthogonal planes of the cell surface morphology as well as the cortical layer and interior cytosol domains, displaying the values of the phase field variables for each domain. In Fig 7E and 7F the model predictions for hydrostatic pressure distributions and stored stress in the same orthogonal planar sections for the stationary morphology are given correspondingly. The pressure values are not unreasonable (e.g. a 1 mm depth of water at atmospheric pressure exerts a hydrostatic pressure of 9.8 Pa). The pressure is low and positive in the external aqueous medium and cytosol; therefore, an inward pressure is exerted from the external medium to the cell surface and an outward pressure from the cell interior (cytoplasm) to the cortex. The pressure is negative in the plasma membrane and cortical layer meaning that this layer experiences an inward compressive pressure from the external aqueous medium and cytosol. The highest (compressive) pressures arise in the cell surface “interphase” nearby high curvature BLiPs, with about an order of magnitude lower values within the cortical layer itself. These stationary pressure gradients suggest a propensity for fluid absorption from the exterior aqueous medium into the cortex phase and cell surface interface. It is important to note that our simulations assume a stationary morphology, and the pressure and stress distributions are a consequence of the stationary assumption. In reality, these morphologies are non-stationary, and in particular there is flow of the cytoplasm that fills the BLiPs. The new balance of pressure and stress will then dictate the directional flux and flow of the external aqueous medium and cytosol through the various cell compartments.

**Fig 8:**
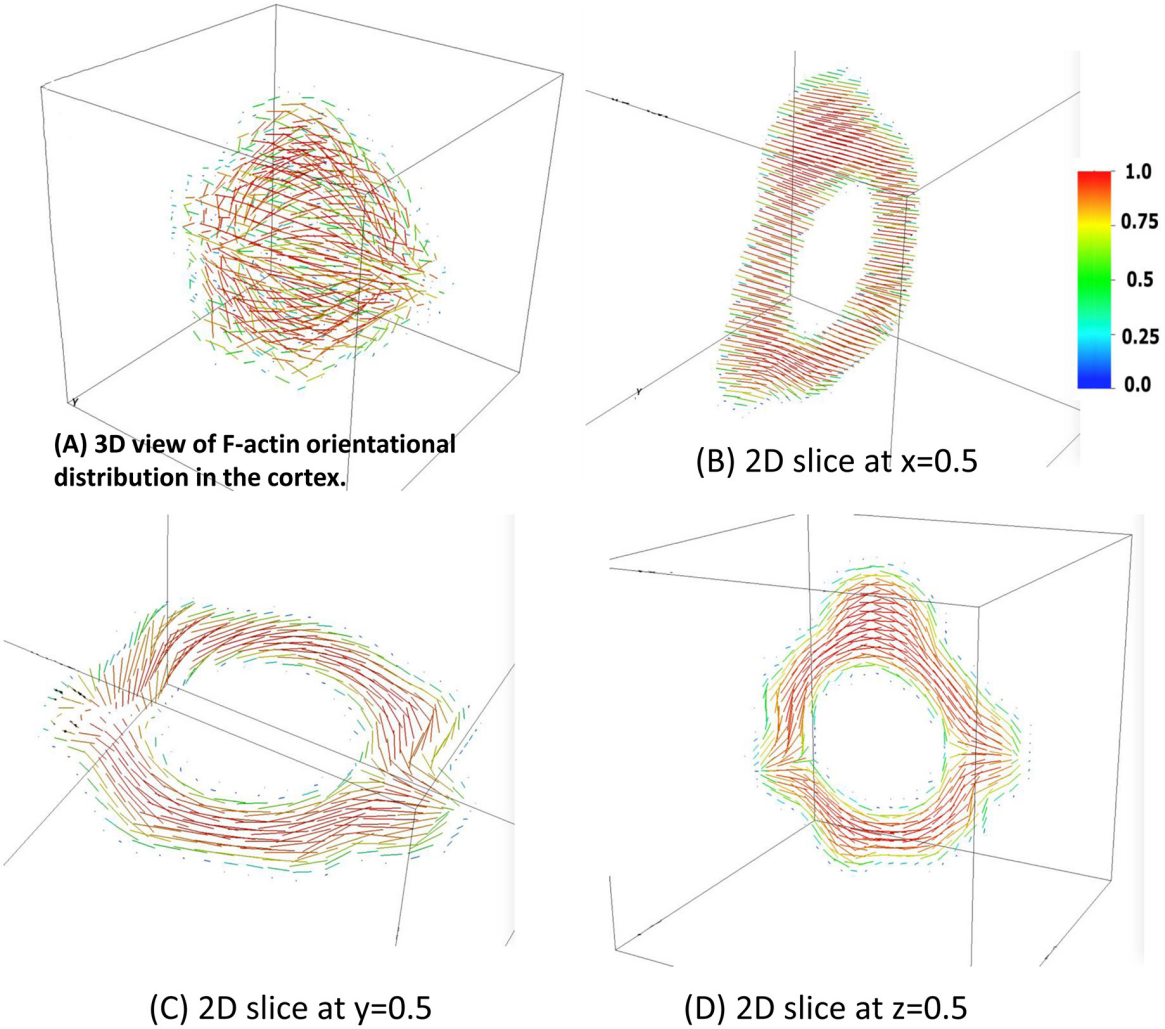
The orientational distribution of F-actin filaments in the nematic cortex for the steady state cell morphology associated with the 3D target morphology of Fig 7. Since the Flory order parameter is unknown, for illustrative purposes we impose its maximum value of 1 in this simulation, and impose lower values in the S6 and S7 Figs. (A) 3D view; (B-D) 2D planar projections of the nematic director field in the cortex in the *x* = 0.5, *y* = 0.5, *z* = 0.5 planes, respectively. The color bar shows the magnitude of nematic director, where |p| = 1 denotes nematic phase and |p| = 0 denotes the isotropic phase.

The 3D orientational distributions of cortical actin-filaments, comprising the nematic cortical phase, are given in Fig 8A; planar 2D slices are shown at the specified positions in Fig 8B–8D. These figures convey the degree and direction of order within the F-actin filaments of the cortex, and their strong correlation with the cell surface morphology. Note that cortical F-actin is assumed to prefer parallel alignment at both cortical interfaces for this illustration of the 3D phase field model. The anchoring energy at the interfaces together with the presumed strength of the nematic potential are responsible for the relatively high degree of alignment of the F-actin; these parameters are tunable to match experimental data on nematic order within the cortex.

The nematic order was changed by varying Flory order parameter from 1 to values approaching 0. The results of these simulations are shown in S6 Fig which shows the nematic order superimposed on the 3D morphology for order parameters that range from 1.0 to 0.01. Cross-sections taken at three orthogonal planes are shown in S7 Fig. Note that the values assumed for K (the Franck elastic constant), and h_1_ and h_2_ will not significantly affect the pressure or the Ericksen stress of the stationary morphology; this is because the dominant contribution to the Ericksen stress is from spatial gradients of the level set function *ϕ*_*1*_ = 1/2 that defines the cell surface. As shown in S6 and S7 Figs, h_1_ and h_2_ only affect the nematic order of the stationary state and K does not affect any of the predictions for the stationary morphology. However, for dynamic processes, these parameter values will strongly dictate the results of the simulation.

## Discussion

Using comparative measurements of many individual cells in two distinct configurations, spread on a substrate versus in a rounded state detached from the substrate, we showed that the cell surface area in the rounded state is highly convoluted and far exceeds the surface area of a sphere that would enclose the volume of the cortex, cytosol and nucleus. We analyzed the size and distribution of bleb-like protrusions (BLiPs) on the cell periphery that served as storage for the excess of surface area. We then developed three complementary modeling approaches that incorporate the concept of excess surface area on rounded cells in different ways.

Employing a 2D discrete geometric model, we tested whether the two-layer composition of the cortex, with the outer layer termed the cell surface, giving the local shape of the BLiPs and the inner layer responsible for contraction, is sufficient to reproduce the highly folded surface observed after rounding. This model demonstrated that morphologies with longer perimeters corresponding to larger ERs will have lower energy compared to the shapes with the same number of folds but with shorter perimeters (i.e. smaller ERs). This result predicts that during cell rounding, larger folds, which are energetically more favorable, should appear early in the process. On the other hand, smaller folds will appear later because it can require time to build structures that will support the higher curvature folds. Indeed, several preliminary experiments in which cells are detached and imaged appear to support this notion. In the early stages of rounding, big folds often appear on the cell surface but, as time goes on, the cell breaks the large folds into the smaller ones. This is supported by the fact that we see a prevalence of small BLiPs in the distribution of fold sizes from the SEM and fluorescence imaging. It is also possible that smaller BLiPs are required as the cell approaches a rounded, steady state because the large folds stored too much of the cell volume (S3D Fig) which could disrupt normal cell functioning. In addition, the ability to create smaller folds would be advantageous in storing large surface excesses in that small BLiPs could form under larger ones for more efficient packing.

Our 3D random “seed and growth” model of BLiPs approximated the BLiP number density and size distribution from 3D scanning electron micrographs. The model provided insight into the SEM image analysis that revealed skewed size distributions of the BLiPs, with a preponderance of small-scale features and successively fewer large-scale protuberances. Moreover, this model demonstrates that efficient packing of BLiPs requires that heterogeneity of BLiP sizes is needed to recapitulate 3D morphologies.

To begin to capture the physical properties underlying the convoluted morphology of the rounded cell, we introduced a generalized phase field formulation. The model accounted for the cell surface as a diffuse interface between the exterior aqueous phase and the interior cortical phase and cytoplasm. In the model, the cell surface is equipped with a Helfrich bending elastic energy that includes a spontaneous curvature function that encodes the bending energy associated with the BLiPs. The spontaneous curvature function is a consequence of molecular components (the spectrin-actin “fishnet”) that mediate the attachment between the cell surface and cortex. However, this molecular information is implicit at this stage of the phase field model, with future extensions aimed at coupling these molecular origins of the spontaneous curvature. At this juncture, our multi-compartment, phase field model accepts 2D or 3D images of the cell morphology as input targets and “learns” the membrane curvature of that target morphology. Since each cell compartment is endowed with constitutive properties, the phase field model predicts physical consequences of the target morphology throughout the cell compartments, restricted for this study to input stationary morphologies. In particular, the model predicts pressure and stress distributions that are concentrated within the cell surface diffuse interface and highly correlated with membrane-cortex interface gradients associated with BLiPs. In future model developments, when the dynamics of the rounded phenotype are introduced, the pressure-stress distributions will evolve in time, and the consequences of constitutive properties of each compartment will dominate the evolution, unlike the stationary predictions where viscous and nematic stresses relax to zero.

How do these three distinct approaches relate to one another? We postulate that cell surface regions rich in adaptor proteins bind the cell surface to the cortex, inheriting the mean curvature of the cortex. We have termed these regions attachment or contact points. Although the species composing these regions have not been identified, presumably they would belong to groups such as the ERM family of cytoskeletal-membrane adaptors as previously suggested in [24]. Moreover, one would expect that these contact points would be transient and regulatable leading to more dynamic behavior than we capture in the current models. Domains with less binding proteins allow the cell surface to detach from the contractile part of cortex forming BLiPs. We assume in our models that the distribution of binding protein species dictates the surface morphology, which in turn dictates the spontaneous curvature function. In the discrete geometric Hamiltonian model, the binding sites forming the attachments between the cell surface and the cortex are explicitly modeled, leading to an induced cell surface morphology.

In the phase field model, we choose the level set *ϕ*_1_ = 0.5 to define and match the surface morphology captured in 2D micrographs or reconstructed in 3D using the seed-and-growth model. Thus discrete Hamiltonian and phase field modeling approaches are complementary: the discrete Hamiltonian model is based on postulated attachment points that determine morphology, whereas the phase field model, in which specific molecular features are coarse-grained, is based on a spontaneous curvature function specific to and constructed from the morphology itself. In 3D, micrographs are not sufficient to provide a 3D image file due to significant occluded cell surface. Thus, we used images produced by seed-and-growth model that yields 3D images of the surface morphology that are statistically consistent with the measured 3D BLiP distribution data from scanning electron microscopy images. The phase field model then uses the 3D surface construction as an imposed target morphology, and the model evolution adapts the spontaneous curvature function until the target morphology is reached. The phase field model then predicts the stationary stresses and energies within the cell surface and cortex self-consistent with that surface morphology. It is important to note the *stationary* aspect of the model predictions which identify stress contributions that are due to spatial gradients of the fitted membrane morphology. Indeed, since the model simulation converges to the input *stationary* morphology, the stored stresses due to nematic elasticity all relax and are negligible. I.e., the stress components are insensitive to the nematic parameters, and are dominated by the gradients of the level set function *ϕ*_1_ = 0.5 learned from the morphology. The power of the model will be further revealed when we investigate the dynamics of the highly convoluted morphology, where nematic parameters and constitutive properties of all compartments will then have significance. It will be important from a biological standpoint to learn the bounds on these parameters.

An additional caveat is that the models presented are purely mechanical or steric in nature. It is certainly possible that active processes other than cortical contraction, giving rise to cortical tension [32] during rounding could play a role even in the short time span of cell rounding from a spread state. For example, in our model, we did not include the actin polymerization process explicitly although but it is true that smaller BLiPs, which are the majority of the population, have higher bending energy so that they require stronger cortical support perhaps requiring additional actin nucleation and polymerization [33] on short time scales.

Highly convoluted surface morphologies are often apparent in three-dimensional tissue contexts and in cells that are not fully spread on a two-dimensional surface. The storage of the cell surface in folds or bleb-like protrusions at the cell periphery is likely to be crucial to a variety of rapid cell shape changes such as those that occur in cell migration. It seems more feasible and energetically favorable that rapid cell shape changes can be accomplished quickly by calling upon and pulling out the excess surface stored in the BLiP distributions as an alternative to large scale endo- and exocytosis accompanied by membrane-cortex remodeling. Although the results presented here are derived for stationary BLiP-laden morphologies, these models form the foundation for future studies of cell surface dynamics regulated by coupling to reaction-diffusion kinetics of various molecular species. These kinetics can be expected to be controlled by signal transduction in many cases. The theoretical approaches presented here should find application in a number of different biological contexts.

## Methods

### Development of the discrete geometric model

The bead-spring model consists of two-layers (Fig 2A), where one layer (outer layer) represents the membrane and underlying actin mesh (i.e. the cell surface) and the other layer (inner layer) represents the myosin-rich contractile cortex. Within each layer, beads are connected pairwise by springs. Special contact points serve to connect the two layers via springs. At the beginning of simulation both layers have the same perimeter. During the simulation the inner layer (cortex) shrinks in order to reach the target enclosed area with smaller perimeter, imitating cortex contraction. The presence of contact points between two layers enforces outer layer bending (S2 Fig). Although the more correct definition of the excess surface ratio is $\varepsilon_{2D}=\frac{\bar{L}}{2\pi \bar{R}}=\bar{L}/2\sqrt{\pi A_{total}}$, where $\bar{L}$ is the perimeter of the surface layer and $\bar{R}$ is the radius of the circle that would enclose the area inside this surface layer (A_total_), for the simplicity we define excess surface as a ratio between the perimeters of surface layer and contracted cortex.

Let the surface layer with the perimeter L be represented by *N* beads (S2 Fig), with the notational convention that bead 0 corresponds to bead *N* (representing a closed contour). Then the Hamiltonian for this outer layer of beads and springs (i.e. the cell surface) is:

$$H_{out}=c_{1}\Sigma_{i=1}^{N}\kappa_{i}^{2}+c_{2}\Sigma_{i=1}^{N}{\left(l_{i}-\bar{L}/N\right)}^{2},$$ (2)

where *k*_*i*_ is the local curvature of the surface at bead *i*; *l*_*i*_ is the length of the spring between beads *i* and *i*+1; and *c*_1_ and *c*_2_ are free parameters that define relative contributions of the energy terms. The first term in Eq 2 is the energy cost for bending the surface layer. The second term ensures that the outer layer does not significantly stretch or contract during the simulated process. *c*_1_ and *c*_2_ are chosen with *c*_2_ ≫ *c*_1_ so that as the system approaches a steady (minimum energy) state, the first term tends to a configuration that minimizes curvature and the second term tends to zero.

The Hamiltonian of the inner, contractile layer (i.e. the cortex) is:

$$H_{inn}=c_{3}\Sigma_{j=1}^{M}p_{j}^{2}+c_{4}{\left(A-\bar{A}\right)}^{2},$$ (3)

where *M* is the number of beads in the inner cortex (*M*<*N*); *p*_*j*_ is the length of the spring between inner beads *j* and *j*+1; *A* is the area of the polygon formed by the inner beads with perimeter *P*; $\bar{A}$ is the target area; and *c*_3_ and *c*_4_ are scaling parameters that define relative contributions of the energy terms. At the steady state this layer approaches the circular shape with $A\to \bar{A}$ and $l_{j}\to 2\sqrt{\pi \bar{A}}/M$.

The total Hamiltonian of the two-layer system contains three additional terms:

$$H_{tot}=H_{out}+H_{inn}+H_{contact}+H_{cross}+H_{self}.$$ (4)

*H*_*contact*_ is the energy stored in the springs between inner and outer cortex contact points:

$$H_{contact}=c_{5}\Sigma_{i=1}^{P}∥t_{i}-\tau_{i}{∥}^{2},$$ (5)

where *t*_*i*_ denote the contact points on the outer cortex and *τ*_*i*_ denote the corresponding contact points on the inner cortex. This term ensures that these contact points remain close. *H*_*cross*_ penalizes crossing of outer cortex beads into the inner cortex polygon:

$$H_{cross}=c_{6}\Sigma_{i=1}^{M}∥t_{i}-\bar{l}∥1_{p_{i}\in Inn},$$ (6)

where *Inn* denotes the interior of the polygon formed by the points of the inner cortex, and the indicator function $1_{p_{i}\in Inn}\;=\;1$ if the outer cortex point *p*_*i*_ is in *Inn* and 0 if outside. *t*_*i*_ denote the points on the outer cortex and $\bar{l}$ denotes the segment closest to each point. We calculate this function by computing the point’s winding number. Lastly, *H*_*self*_ penalizes self-crossing of the outer polygon. Let $\bar{l}$ denote the line segment connecting beads *i* and *i*+1.

$$H_{self}=c_{7}\Sigma_{i\neq j}cross\left(\bar{l_{i}},\bar{l_{j}}\right),$$ (7)

where the function $cross\left(\bar{l_{i}},\bar{l_{j}}\right)=1$ if $\bar{l_{i}}$ and $\bar{l_{j}}$ cross and 0 if they do not. We let *c*_7_ = ∞ with the convention 0∙∞ = 0, effectively preventing any self-crossings of the outer cortex. In practice, this condition is enforced by considering all other energy terms and keeping bead *i* fixed if $\bar{l_{i}}$ crosses any $\bar{l_{j}}$ for any *j* ≠ *i* in the next iteration. As the system approaches steady state each of these additional terms tends to zero.

While the target area ($\bar{A}$) constraint is more aptly applied to the outer layer, it is numerically more feasible to apply the target area constraint to the inner layer Hamiltonian and to scale the final simulated result by multiplying the coordinates of each point by a multiplicative factor to match the target area. With this scaling the overall shapes of the “cell surface” and cortex do not change but all simulated shapes get the same area inside their surface layer which includes cortex and folds.

### The phase field approach

We introduce phase variables *ϕ*_*i*_,*i* = 1,2,3 (Fig 5) that denote the volume fractions of phase 1 (the exterior aqueous medium surrounding the rounded cell), phase 2 (cortex) and phase 3 (interior cytosol), respectively. Clearly, in any pure phase i, the respective *ϕ*_*i*_ = 1, whereas in diffuse interfaces between phases i and j, *ϕ*_*i*_+*ϕ*_*j*_ = 1, with *ϕ*_*k*_ = 0,*k* ≠ *i*,*j*, and everywhere the total volume fraction is 1. Thus in the external aqueous medium, *ϕ*_1_ = 1; in the F-actin rich, cell cortical layer, *ϕ*_2_ = 1; and in the interior cytoplasm, *ϕ*_3_ = 1. The phase boundaries are: the cell surface, as defined above, that separates the external aqueous medium and cortical layer, where 0<*ϕ*_1_,*ϕ*_2_<1; and, the transition layer between the cortex and interior cytosol where 0<*ϕ*_2_,*ϕ*_3_<1. For graphical purposes and for matching 2D TEM and 3D simulated topology images from the seed and growth model, the cell surface is defined by the level sets *ϕ*_1_ = *ϕ*_2_ = 0.5, while the cortex-cytosol interface is defined by *ϕ*_2_ = *ϕ*_3_ = 0.5. We do not allow all three phases to come into contact in this model, achieved by an energy penalty term. Therefore the level set *ϕ*_1_ = 0.5, in domains where *ϕ*_3_ = 0, determines the cell surface. Below, we illustrate how to constrain this level set function to match the experimentally measured cell surface, in both shape and surface area.

We note that for this paper the external aqueous medium and interior cytosol are modeled as viscous fluids with specified viscosities and the cortex is modeled as a nematic (liquid crystal) gel [31]. Viscoelasticity of the interior cytoplasm is easily incorporated into our phase field formulation [34], but for the purposes of the stationary morphology any stored elastic stress in the cell interior relaxes to zero. Thus we simplify to a viscous cytosol for this paper.

The governing equations for the three phases and two diffuse interfaces are presented next. The phase field method is an energy-based variational theory, comprised of free energy functionals for each phase and diffusive interface.

#### Free energy

We denote the free energy of the cortex by *F*_**p**_, where the subscript **p** is the nematic director; the free energy for all interfacial tensions by *F*_*S*_, where *S* denotes surface energies; and the free energy for the cell surface bending energy by *F*_*B*_, where *B* denotes bending. The cell surface and the F-actin cortex may be bound or tethered, modeled by an orientational anchoring condition that can be tuned between parallel and normal alignment of the cortex, and with an energy cost of membrane-cortex anchoring denoted by *F*_*anch*_. The membrane surface area and the cell volume are assumed to be known from experimental measurements of the spread cell configuration and conserved in the transition from spread to rounded configuration as discussed in the experimental section. The phase transport equations for the phase variables in this paper are Cahn-Hilliard equations that ensure the conservation of volume of each component and thereby conserve cell volume [18] [35]. Therefore, cell volume is encoded in the initial conditions and preserved in all simulations. To ensure conservation of membrane surface area, we introduce an energy *F*_*SA*_ that penalizes the departure from the prescribed, measured, membrane surface area. Putting these contributions together, the total free energy is given by the sum:

$$F=F_{S}+F_{B}+F_{p}+F_{anch}\text{​+​}{\text{F}}_{SA}.$$ (8)

We now describe these energy terms in more detail. The interfacial surface energy contributions are built into *ϕ*_1_ and *ϕ*_2_ at the external aqueous medium-cortex boundary (the cell surface) and into *ϕ*_2_ and *ϕ*_3_ at the cortex-cytosol phase boundary. Each contribution is modeled by a standard phase field approximation to the surface energy at the interface, consisting of an energy penalty for conformational entropy together with the Ginzburg-Landau double well potential whose two minima define the two adjacent phases, and finally an energy term that penalizes coexistence of the three phases:

$$F_{S}=\int_{\Omega }\sum_{i=1}^{3}3\sqrt{2}\gamma_{is}\left(\frac{\varepsilon }{2}∥\nabla \phi_{i}{∥}^{2}+\frac{1}{\varepsilon }\phi_{i}^{2}{\left(1-\phi_{i}\right)}^{2}\right)+\frac{\gamma_{123}}{2}\Pi_{i=1}^{3}\phi_{i}^{2}dx,$$ (9)

where *γ*_1s_ and *γ*_2s_ contribute the cell surface tension while *γ*_2s_ and *γ*_3s_ contribute surface tension for the cortex-cytosol diffuse interface. Here *γ*_123_ is a parameter to penalize coexistence of the three phases.

Since the membrane “surface” is represented by *ϕ*_1_ = 0.5 *ϕ*_2_ = 0.5, the bending elastic energy *F*_*B*_ of the cell surface is built into *ϕ*_1_, given by

$$F_{B}=\int_{\Omega }3\sqrt{2}\gamma_{1b}\varepsilon {\left(\nabla^{2}\phi_{1}-\frac{2}{\varepsilon^{2}}\phi_{1}(\phi_{1}-1)(2\phi_{1}-1-\frac{\varepsilon }{\sqrt{2}}C_{1})\right)}^{2}dx,$$ (10)

where *γ*_1b_ parameterizes the bending rigidity of the bilayer membrane, and *ε* parameterizes the interfacial thickness. The function *C*_1_ is the spontaneous curvature of the cell surface, a key element of our model that warrants discussion. *C*_1_ is a proxy for the heterogeneous “fishnet” or membrane skeleton coupled to the plasma membrane *per se*, as discussed above, that will be explicitly represented by attachments between the plasma membrane and subjacent fishnet as discussed in the discrete geometric model presented above. Regions with a relatively dense fishnet bind the membrane to the cortex, inheriting the (relatively low) mean curvature of the cortex. Regions with a relatively dilute fishnet allow the cell surface to detach from the cortex and in these domains, we surmise that BLiPs form. In the absence of detailed molecular knowledge of the fishnet structure, we use either 2D micrographs of the cell surface morphology or 3D simulations of the cell surface morphology (see the next paragraph) to instead construct the fishnet proxy, the spontaneous curvature function *C*_1_. Note that the interplay between domains poor versus rich in the cell surface-cortex skeleton dictates the surface morphology, which in turn dictates the spontaneous curvature function *C*_1_. We choose the level set *ϕ*_1_ = 0.5 to define and match the membrane morphology captured in 2D micrographs and reconstructed from 3D micrographs.

The elastic energy associated with the (apolar) nematic gel model of the filamentous actin cortex is described in terms of a direction **p** for the principal axis of orientation, while |**p**| is allowed to vary between 0 for the isotropic phase and 1 for a perfectly aligned phase. (We have chosen to use a generalized Leslie-Ericksen-Oseen-Frank model of the nematic cortex in this study, rather than more complex models based on the second moment tensor of the F-actin orientational distribution. The rationale is both for modeling simplicity and to minimize the set of constitutive parameters for the cortex that require experimental measurement. The vector with direction **p** is the nematic director and |**p**| is the scalar order parameter. The elastic energy of the cortex is then given by a Frank-Oseen distortional energy together with a Landau-deGennes bulk free energy,

$$F_{p}=\int_{\Omega }\frac{1}{2}\phi_{2}^{2}\left(\frac{K}{2}∥\nabla p{∥}^{2}+\frac{h_{2}}{4}∥p{∥}^{4}-\frac{h_{1}}{2}∥p{∥}^{2}\right)dx,$$ (11)

where the prefactor $\phi_{2}^{2}$ restricts the nematic elastic energy to the cortex, *K* is the Frank elastic constant (we assume the bend, splay and twist constants are equal in this paper), controlling energy cost for orientational gradients within the actin-rich cortical network; and *h*_1_,*h*_2_ are model parameters that control whether the equilibrium phase of the cortex is nematic (|**p**| ≠ 0) or isotropic (|**p**| = 0). In a spatially homogeneous state, where ∇**p** vanishes, the elastic energy for the cortex favors stable minima of the bulk energy function: *h*_2_/4‖**p**‖^4^−*h*_1_/2‖**p**‖^2^. For *h*_2_>0, the stable minimizer of this function is given by the nematic state $\left|\text{p}\right|=\sqrt{h_{1}/h_{2}}$ when *h*_1_>0 and by the isotropic state **p** = 0, when *h*_1_≤0.

The anchoring energy of the nematic cortex with the cell membrane is given by

$$F_{anch}=\int_{\Omega }\frac{\alpha_{1}}{2}{\left(p\cdot \nabla \phi_{1}\right)}^{2}dx,$$ (12)

where *α*_1_ parameterizes the strength of the anchoring potential, and *α*_1_>0 promotes tangential anchoring while *α*_1_<0 promotes normal anchoring of the F-actin cortical network with the cell surface.

The target surface area is *s*_0_S*, where S* is the initial external surface area of the plasma membrane (the rounded base morphology) and *s*_0_ is the excess surface area ratio. The excess membrane surface is enforced by an energy penalty for deviation of the simulated, model surface area *S*(*t*) from the imposed target surface area. The energy penalty function is given by

$$F_{SA}=\frac{1}{2}\lambda_{S}{\left(S\left(t\right)-s_{0}S^{*}\right)}^{2}$$ (13)

where *λ*_*S*_ weights this energy penalty relative to other energy contributions, and *S(t)* can be estimated from the phase variable ϕ_1_ by an integral over the entire computational domain (see [36,37] for the rigorous argument):

$$S\left(t\right)=\int_{\Omega }3\sqrt{2}\left(\frac{\varepsilon }{2}\|\nabla \phi_{1}{\|}^{2}+\frac{1}{\varepsilon }\phi_{1}^{2}{\left(1-\phi_{1}\right)}^{2}\right)dx$$ (14)

We note that this variational energy model accepts the measured target for *s*_0_S*, and the microscopic images are used to fit *C*_*1*_ from 2D cell perimeters or 3D cell surface reconstructions. In this way, any initial guesses for S follow the energy minimization dynamics toward the target surface area, while *C*_*1*_ guides the cell surface morphology toward that of the experimental micrographs.

With the free energy functional for the multiphase cell and external aqueous medium outlined above, a hydrodynamic phase field model is then derived. The details of the governing system of equations, the numerical method, and the parameter values we employ in simulations are deferred to S2 Appendix.

### Cell culture

Swiss 3T3 cells (obtained from Tissue culture facility UNC Chapel Hill) were cultured in DMED (Gibco) with 10% FBS(Gibco). CHO-wt cells (from ATTC) were grown in medium DMEM/F12 (Gibco) containing 10% FBS. CHO cells stably expressing Lifeact-GFP (the small 17-amino-acid peptide,Lifeact, fused to green fluorescent protein, GFP) were obtained from the James Bear laboratory (UNC-Chapel Hill). CHO-wt cells were transiently transfected by GFP-PH-delta domain (gift from Con Beckers, UNC-CH) using Lipofectamine Plus reagent (Invitrogen) and images were taken 24–48 hours after transfection.

### Imaging

#### Light microscopy

For visualization of cell rounding and estimation of cell sizes, cells were grown for 24 h on glass bottom dishes. At the beginning of experiment cells were imaged in spread state and then were detached by trypsin under continuous microscope recording. After the cells rounded, medium was added to the dish cells were imaged. Images were acquired with a 60x oil immersion objective using an Olympus FluoView1200 laser scanning confocal microscope employing an environmental chamber.

#### Scanning electron microscopy

CHO cells were grown in cell culture flask and on two coverslips for 24h. Cells from flask were detached by trypsin, spun down, resuspended in 2 mL of culture media. 1 mL of resuspended cells was added to one of the coverslips with spread CHO cells (50% confluency) and incubated for 20 min. Another coverslip containing spread CHO cells was treated with trypsin under microscope observation to insure that the cells rounded but did not completely detach. Both coverslips were fixed with a solution of 2.5% glutaraldehyde/HBSS, pH 7.4, for one hour at room temperature. Following three rinses with 0.15M sodium phosphate buffer, pH 7.4 (PB), the cells were post-fixed in 1% osmium tetroxide in PBS for 30 minutes followed by subsequent treatment with 2% tannic acid for 10 minutes and 1% osmium tetroxide in water for 10 minutes. The coverslips were dehydrated with ethanol (30%, 50%, 75%, 100%, 100%), transferred to a Samdri-795 critical point dryer and dried using carbon dioxide as the transitional solvent (Tousimis Research Corporation, Rockville, MD). Coverslips were mounted on aluminum planchets with double-sided carbon adhesive and coated with 10nm of gold-palladium alloy (60Au:40Pd, Hummer X Sputter Coater, Anatech USA, Union City, CA). Images were taken using a Zeiss Supra 25 FESEM operating at 5kV, working distance of 5mm, and 10μm aperture (Carl Zeiss SMT Inc., Peabody, MA).

#### Transmission electron microscopy

CHO cells stably expressing Lifeact-GFP were grown to confluency, detached by trypsin, spun down, resuspended in cell culture medium, transferred to a glass bottom dish and incubated for 20 min. Cells were fixed with 4% paraformaldehyde/HBSS and then underwent osmium treatment, dehydration, and resin infiltration steps with coverslip separation. For immunoelectron microscopy, primary antibody (Cell Signaling #2956 (GFP[D5.1]XP Rabbit mAb) and secondary goat anti-rabbit conjugated to 5nm or 15nm colloidal gold diluted 1:100 was employed. After preliminary observation, 5nm Au-labeled grids were enhanced with Nanoprobes Gold Enhance (#2113, Nanoprobes, Yaphank, NY) reagent for 3 minutes. Stained sections were observed using a LEO EM910 electron microscope operating at 80kV (Carl Zeiss SMT, Inc., Peabody, MA) and photographed using a Gatan Orius SC1000 CCD Digital Camera and Digital Micrograph 3.11.0 (Gatan, Inc., Pleasanton, CA).

## Supporting Information

S1 Appendix The possible surface excess that can be stored on the rounded cell with equally sized sphererical BLiPs. (PDF)

S2 Appendix The governing system of equations, the numerical method, and the parameter values for phase field simulations. (PDF)

S1 Fig Fluorescence and DIC imaging of rounded CHO cell stably expressing RFP-Myosin and GFP-Lifeact. Images taken along Z direction from cell bottom with 1um step.
(PDF)

S2 Fig Schematic of the computational progression from initial (*i)* to final state (*iii)* for the geometric model. *i*.) Initial condition: both the cell surface perimeter, L, and cortex perimeter, P_initial_, start out at identical, coincident positions enclosing area A. N contact points are equally distributed around the perimeter. The inner yellow circle represents the target area, Atarget, to which the cortex will shrink during the computation, imitating a two-dimensional cell rounding. *ii*) A snapshot at the early stages of computation when inner layer already shrunk to target area with perimeter, P_final_. Because the inner and outer layers are connected via contact points, folds(BLiPs) are formed around the 2D cell periphery but bending energy of folds is not minimized yet. *iii*) The final steady state is achieved by minimizing the Hamiltonian (in effect, the curvature of the outer, cell surface layer). In this configuration, contact points on the outer and inner layer meet and, because curvature energy is minimized, the system ceases to further evolve.
(PDF)

S3 Fig A. Three overlapping shapes, each has the same area and initial excess ratio ER = 4. The shapes were prescribed to have different number of folds: 20 (yellow), 40 (red), 50 (blue). The blue shape has smaller local radius along the perimeter which results in higher bending energy. B. Three overlapping shapes, each has the same area and number of folds, N = 40. The shapes have different initial excess ratio: 6 (yellow), 4 (red), 2 (blue). The blue shape has smaller local radius along the perimeter which results in higher bending energy. C. Plot of bending energy for simulated shapes with different ERs and number of folds. D. Plot of area stored in BLiPs for different shape configurations.
(PDF)

S4 Fig A SEM image of CHO cells. Part of cells was spread on glass bottom dish for 24 hours and another part was detached and plated on the same dish 20min before fixation. Bar = 10.
(PDF)

S5 Fig Estimation of BLiPs sizes. A. SEM image of rounded CHO cells. B. Enlarged part of image on A (yellow dotted line) with some BLiPs contoured as example of how radius was estimated. Using ImageJ the area within each contour was measured and the radius was calculated assuming that the measured area represents a great circle of BLiP. C. Distribution of BLiPs radii of all cells on image A estimated as it shown on B.
(PDF)

S6 Fig(a) shows the basic cell morphology. The black lines in (b)-(e) denote the direction and magnitude of the nematic director, **p** where |p| indicates the degree of order between 0 (isotropic) and 1 (maximum order). Here $\sqrt{h_{1}/h_{2}}$ prescribes the Flory order parameter of the nematic equilibrium. Here we choose h1 = 2x102N/m2 and h2 = 2 x 102, 8 x 102, 2 x 104, 2 x 106 N/m2, for (b)-(e) respectively.(PDF)

S7 Fig2D slices of the surface morphology and director orientation from S6 Fig.(a-c) $\sqrt{h_{1}/h_{2}}$ = 1; (d-f) $\sqrt{h_{1}/h_{2}}$ = 0.5; (g-i) $\sqrt{h_{1}/h_{2}}$ = 0.1. The values of X, Y & Z indicate the position of the 2D slice on the respective axes.(PDF)
