# Supplementary material for: Modeling the Excess Cell Surface Stored in a Complex Morphology of Bleb-Like Protrusions
Source: PLoS Comput Biol. 2016 Mar 25;12(3):e1004841. doi: 10.1371/journal.pcbi.1004841 (PMC4807848; doi:10.1371/journal.pcbi.1004841)
Supplement: S1 Appendix — (PDF) [file pcbi.1004841.s001.pdf]

S1 Appendix for "Modeling the excess cell surface stored in a complex morphology of bleb-like protrusions"

# The possible surface excess that can be stored on the rounded cell with equally sized sphererical BLiPs.

## 1 Membrane excess

Suppose a *spread* cell has volume $V_0$ and membrane surface area $S_0$. We define membrane excess as $\varepsilon = \frac{S_0}{S}$ , where $S$ is the surface area of a sphere with volume $V_0$. Thus,

$$\varepsilon = \frac{S_0}{4\pi}\left(\frac{4\pi}{3V_0}\right)^{2/3} \quad (1)$$

## 2 Two dimensional case

In 2D, the definition of membrane excess becomes

$$\varepsilon = \frac{P_0}{2\pi}\sqrt{\frac{\pi}{A_0}} \quad (2)$$

Let's assume that a *rounded* cell has circular folds ("blisters") of radius $r$ tightly covering the cell body of radius $R$ (Fig. 1). Then,

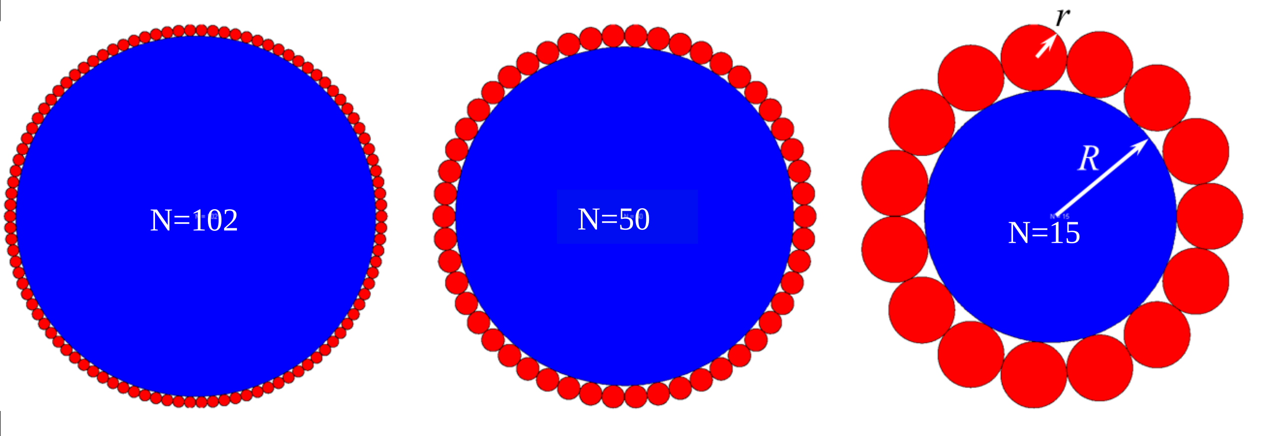


Fig 1

Total area: $$\pi R^2 + N\pi r^2 = A_0 \quad (3)$$

Total perimeter: $$2\pi R + N2\pi r = P_0 = 2\varepsilon\sqrt{\pi A_0} \quad (4)$$

where the number of folds is $$N = \frac{\pi}{\arcsin\left(\frac{r}{R+r}\right)} \tag{5}$$

In the limit $r << R$,

$$N \approx \frac{\pi R}{r} \tag{6}$$

and

$$\pi R^2 + \pi^2 r R = A_0$$
$$\pi R + \pi^2 R = \varepsilon\sqrt{\pi A_0}$$

so that

$$R = \frac{\varepsilon\sqrt{A_0}}{\sqrt{\pi}(1+\pi)} \tag{7}$$

$$r = \frac{\sqrt{A_0}}{\pi\sqrt{\pi}}\left(\frac{1+\pi}{\varepsilon} - \frac{\varepsilon}{1+\pi}\right) \tag{8}$$

$$N = \frac{\pi^2\varepsilon^2}{(1+\pi)^2 - \varepsilon^2} \tag{9}$$

Therefore, membrane excess can be accounted in the given geometry $(r > 0)$ if

$$\boxed{\varepsilon < (1+\pi)} \tag{10}$$

Since the integrated curvature of a circle is $2\pi r (1/r)^2 = \frac{2\pi}{r}$, the total curvature is

$$K = \frac{2\pi}{R} + N\frac{2\pi}{r} \approx \frac{2\pi}{R} + \frac{2\pi^2 R}{r^2} \xrightarrow[r\to 0\,(\varepsilon\to 1+\pi)]{} \infty$$

## 3 Three dimensional case

In 3D, (3)-(10) become

Total volume: $$\frac{4}{3}\pi R^3 + N\frac{4}{3}\pi r^3 = V_0 \tag{11}$$

Total surface area: $$4\pi R^2 + N 4\pi r^2 = S_0 = 4\pi\varepsilon\left(\frac{3V_0}{4\pi}\right)^{2/3} \tag{12}$$

The number of folds (for $r << R$): $$N \approx \frac{4\pi R^2}{\pi r^2} = 4\frac{R^2}{r^2} \tag{13}$$

From (11)-(13) we find that

$$R = \sqrt{\frac{\varepsilon}{5}} \left( \frac{3V_0}{4\pi} \right)^{1/3} \quad (14)$$

$$r = \frac{1}{4} \left( \frac{3V_0}{4\pi} \right)^{1/3} \left[ \frac{5}{\varepsilon} - \sqrt{\frac{\varepsilon}{5}} \right] \quad (15)$$

$$N = \left[ \frac{8\varepsilon\sqrt{\varepsilon}}{5\sqrt{5} - \varepsilon\sqrt{\varepsilon}} \right]^2 \quad (16)$$

In this case, membrane excess can be accounted in the given geometry $(r > 0)$ if

$$\boxed{\varepsilon < 5}$$

**Numerical example:**

Fig. 2 shows the dependence of $R(\varepsilon)$, $r(\varepsilon)$ and $N(\varepsilon)$, according to equations (7)-(9) for $A_0 = 570\ \mu m^2$ and equations (14)-(16) for $V_0 = 10000\ \mu m^3$. Grey area corresponds to $r$ between 0.5 and 1.0 $\mu m$.

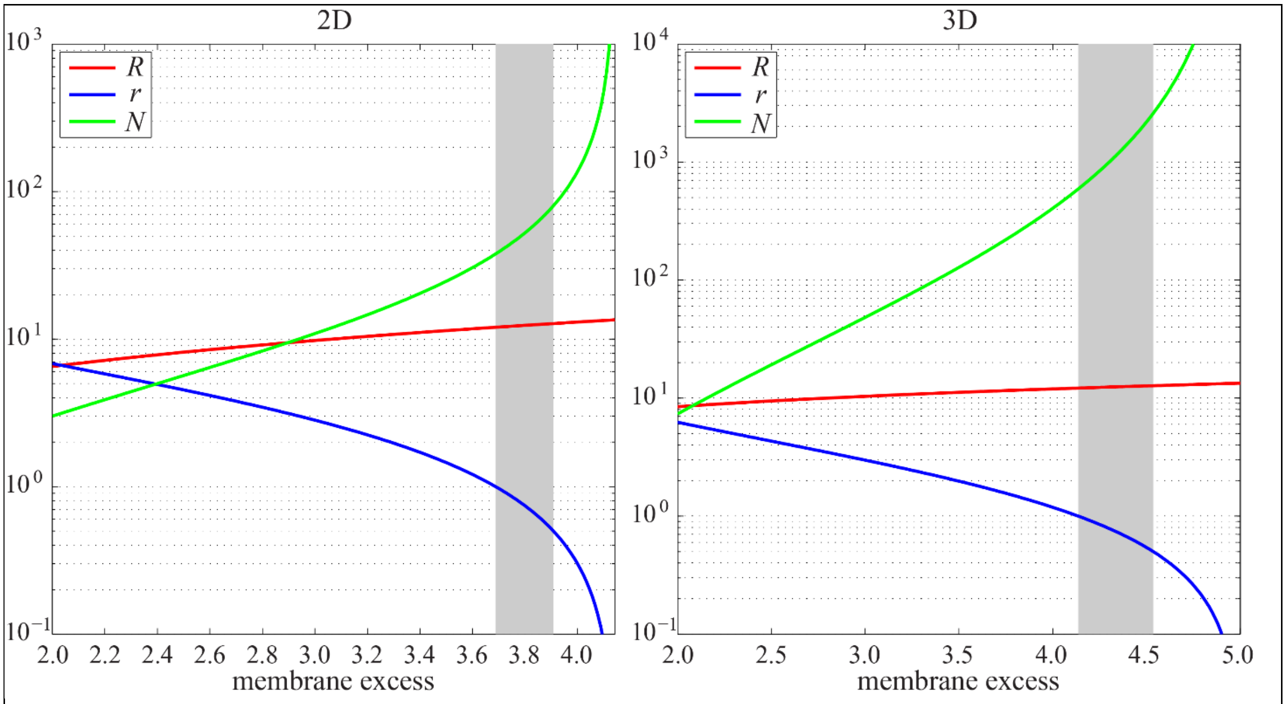


Fig. 2

**Comment:** the above arithmetic is valid only under assumption that the folds are circular (spherical) with the same size. For such geometry the maximum membrane excess is $1+\pi$ (in 2D) and 5 (in 3D). In different geometry, such as sinusoidal folds (Fig. 3), the membrane excess doesn't have to be bound. However, this geometry requires higher integrated curvature. We can

use our discrete model, that minimizes curvature but is not limited to a specific geometry, to explore different shapes as a function of membrane excess $\varepsilon$ and the number of folds $N$.

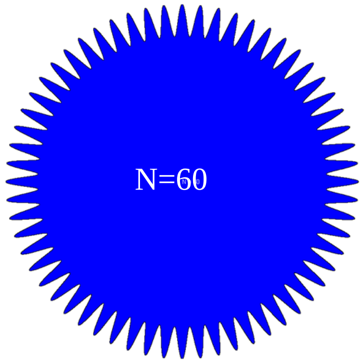

Fig. 3
