# Supplementary material for: Modeling the Excess Cell Surface Stored in a Complex Morphology of Bleb-Like Protrusions
Source: PLoS Comput Biol. 2016 Mar 25;12(3):e1004841. doi: 10.1371/journal.pcbi.1004841 (PMC4807848; doi:10.1371/journal.pcbi.1004841)
Supplement: S2 Appendix — (PDF) [file pcbi.1004841.s002.pdf]

Supplemental material for "Modeling the excess cell surface stored in a complex morphology of bleb-like protrusions"

1 The full set of coupled model equations

By postulating the linear momentum balance and incompressibility of the material system, the governing system of equations is (recall the description of the total free energy F in the main text):

$$\begin{cases} \rho(\partial_t \mathbf{v} + \mathbf{v}\cdot\nabla\mathbf{v}) = -\nabla p + \nabla\cdot\sigma, \\ \nabla\cdot\mathbf{v} = 0, \\ \partial_t\phi_i + \nabla\cdot(\mathbf{v}\phi_i) = \nabla\cdot(\lambda_i\nabla(\mu_i - \mu_2)), \quad i = 1,3. \\ \partial_t\mathbf{p} + \mathbf{v}\cdot\nabla\mathbf{p} - \mathbf{W}\cdot\mathbf{p} = \nu\mathbf{D}\cdot\mathbf{p} + \lambda_p^{-1}\mathbf{h}, \end{cases} \tag{1}$$

where $\mathbf{h} = -\frac{\delta F}{\delta \mathbf{p}}$ is known as the molecular field in the liquid crystal community [3], representing a torque generated by the Frank-Oseen elastic energy, $\mu_i = \frac{\delta F}{\delta \phi_i}$ is the chemical potential with respect to ϕ_i, given by

$$\begin{aligned} \mu_3 &= 3\sqrt{2}\gamma_{3s}\varepsilon\left(-\nabla^2\phi_3 + f_s'(\phi_3)\right) + \gamma_{123}\phi_1^2\phi_2^2\phi_3, \\ \mu_2 &= 3\sqrt{2}\gamma_{2s}\varepsilon\left(-\nabla^2\phi_2 + f_s'(\phi_2)\right) + \gamma_{123}\phi_1^2\phi_2\phi_3^2 + \phi_2\Big(\tfrac{K}{2}(\nabla\mathbf{p})^2 + \tfrac{h_2}{4}|\mathbf{p}|^4 - \tfrac{h_1}{2}|\mathbf{p}|^2\Big), \\ \mu_1 &= 3\sqrt{2}\gamma_{1s}\varepsilon\left(-\nabla^2\phi_1 + f_s'(\phi_1)\right) + \gamma_{123}\phi_1\phi_2^2\phi_3^2 \\ &\quad +3\sqrt{2}\gamma_{1b}\Big(\nabla^4\phi_1 - f_b'(\phi_1)\nabla^2\phi_1 - \nabla^2 f_b(\phi_1) + f_b'(\phi_1)f_b(\phi_1)\Big) - \alpha_1\nabla\cdot((\mathbf{p}\cdot\nabla\phi_1)\mathbf{p}), \\ \mathbf{h} &= -K\nabla\cdot(\tfrac{\phi_2^2}{2}\nabla\mathbf{p}) + \tfrac{\phi_2^2}{2}(-h_1 + h_2|\mathbf{p}|^2)\mathbf{p} + \alpha_1(\mathbf{p}\cdot\nabla\phi_1)\nabla\phi_1, \end{aligned} \tag{2}$$

with $f_s(\phi) = \frac{1}{\varepsilon^2}\phi^2(1-\phi)^2$ and $f_b(\phi) = \frac{1}{\varepsilon^2}\phi(\phi-1)(2\phi - 1 + \frac{\varepsilon}{\sqrt{2}}C_1)$, , where C_1 is the spontaneous curvature functional defined in the main text and approximated from the phase field variable ϕ_1 by (6) below. Here σ is the total extra stress, $\mathbf{W}_{\alpha\beta} = \frac{1}{2}(\partial_\beta\mathbf{v}_\alpha - \partial_\alpha\mathbf{v}_\beta)$ is the vorticity tensor, $\mathbf{D}_{\alpha\beta} = \frac{1}{2}(\partial_\beta\mathbf{v}_\alpha + \partial_\alpha\mathbf{v}_\beta)$ is the rate of strain tensor, ν is a geometric parameter for the nematic gel, and λ_p is a rotational relaxation time for the nematic director $\mathbf{p}$. Here the total extra stress tensor consists of three parts:

$$\sigma = \sigma^r + \sigma^d + \sigma^e, \tag{3}$$

where σ^r is the elastic stress corresponding to the motion of the nematic director $\mathbf{p}$, σ^d is the viscous stress associated to the solvent in the system, σ^e is the Ericksen stress, the stress associated to the elastic interfacial force due to molecular convection. They are given specifically by the following:

$$\begin{aligned} \sigma^r &= -\tfrac{\nu}{2}(\mathbf{ph} + \mathbf{hp}) + \tfrac{1}{2}(\mathbf{ph} - \mathbf{hp}), \\ \sigma^d &= 2\eta\mathbf{D}, \\ \sigma^e_{\alpha\beta} &= (f - \textstyle\sum_{i=1}^3 \phi_i\mu_i)\delta_{\alpha\beta} - \textstyle\sum_{i=1}^3 \frac{\partial f}{\partial(\partial_\beta\phi_i)}\partial_\alpha\phi_i - \frac{\partial f}{\partial(\partial_\beta\mathbf{p}_\gamma)}\partial_\alpha\mathbf{p}_\gamma, \end{aligned} \tag{4}$$

where η is the volume-averaged viscosity, $\eta = \sum_{i=1}^{3} \phi_i \eta_i$, with η_1 the buffer viscosity, η_2 the cortical viscosity and η_3 the cytosol viscosity. The divergence of the Ericksen stress yields the interfacial forces $\nabla \cdot \sigma^e = -(\nabla \mathbf{p}) \cdot \mathbf{h} - \sum_{i=1}^{3} \phi_i \mu_i$.

2 How to fit the spontaneous curvature

Given the TEM (2d) micrograph, we convert the experimental image into gray scale, where each pixel is represented by a number between 0 and 1 that represents the volume fraction of pure buffer, denoted by ϕ_1 (1 represents pure buffer, 0 represents cytosol and cortex). The plasma membrane is the level set $\phi_1 = \frac{1}{2}$. Then, the figure is interpolated into either 256*256 pixels, or 128*128 pixels, depending on the mesh size in the simulation. In a similar manner, for the 3D case, we use the data generated by the seed-and-grow model. The data is stored in either a 256^3 or 128^3 matrix, with each data point between 0 and 1, representing the volume fraction of pure buffer. All data are in Cartesian coordinates.

Denote the unit outward normal vector of the plasma membrane $\{\phi_1 = \frac{1}{2}\}$ as $\mathbf{n}$, which is defined within the phase field context by

$$\mathbf{n} = -\frac{\nabla \phi_1}{|\nabla \phi_1|}. \tag{5}$$

Then the interfacial curvature can be expressed as a function of the phase field variable ϕ_1 via [15]

$$C_1 = -\nabla \cdot \mathbf{n} \approx \frac{1}{|\nabla \phi_1|} \left(\nabla^2 \phi_1 - \frac{\phi_1(1-\phi_1)(1-2\phi_1)}{\varepsilon^2} \right). \tag{6}$$

3 Table of Model Parameters

All parameters are summarized in Table 1, including references that cite the order of magnitude for some, whereas the others are estimated.

Table 1: dimensional and dimensionless parameters

Symbol	Description	Nominal value	Unit	Reference and Remarks
d	Characteristic length scale	1×10^{-5}	m	[14]
t_0	Characteristic time scale	1	s	[14]
ρ	Cell density	1.1×10^3	kg/m^3	[5]
η_1, η_2, η_3	Averaged viscosity	10.0	$N \cdot s/m^2$	$10^{-3} - 1$ [14] for cytosol,100 [8] for cortex
$\gamma_{is}, i = 1, 2, 3$	Surface tension for the interface	5×10^{-5}	N/m	[10]
γ_{1b}	Bending rigidity of cell membrane	1×10^{-18}	$N \cdot m$	[13]
ε	Thickness of the interface	5×10^{-7}	m	model parameter
λ_1, λ_3	Motility parameter	1×10^{-8}	$m^3 \cdot s/kg$	model parameter
λ_p	Time relaxation for the nematic director $\mathbf{p}$	1×10^3	s	[4]
s_0	Excess surface area ratio	3		experiment measured
λ_S	Lagrange multiplier for excess surface area constraint	2×10^5	N/m^3	model parameter
K	elastic strength for cell cortex (Frank elastic constants)	10^{-11}	N	[16, 9]
h_1, h_2	LandauDe Gennes nematic potential parameters	2×10^2	N/m^2	model parameter
α_1	Parallel anchoring strength	10^{-11}	N	assume the same with K
ν	Nematic director tumbling parameter	1.2		rod-like flow-aligning regime

4 Numerical Schemes

We now present the semi-discrete numerical scheme as follows.

Given the initial conditions ($\mathbf{p}^0$, ϕ_1^0, ϕ_2^0, ϕ_3^0, $\mathbf{v}^0 = 0$, $p^0 = 0$), having computed ($\mathbf{p}^n$, ϕ_1^n, ϕ_2^n, ϕ_3^n, $\mathbf{v}^n$,p^n) for $n \geq 0$, we compute ($\mathbf{p}^{n+1}, \phi_1^{n+1}, \phi_2^{n+1}, \phi_3^{n+1}, \mathbf{v}^{n+1}, p^{n+1}$) in the following sequence.

1. Step 1: update $\mathbf{p}^{n+1}$:

$$\left\{\begin{array}{l} \frac{\mathbf{p}^{n+1}-\mathbf{p}^n}{\delta t} + \mathbf{v}^n \cdot \nabla \mathbf{p}^n - \frac{1}{2}(\mathbf{v}^n - (\mathbf{v}^n)^T) \cdot \mathbf{p}^n - \frac{\nu}{2}(\mathbf{v}^n + (\mathbf{v}^n)^T) \cdot \mathbf{p}^n = -\frac{1}{\lambda_p}\mathbf{h}^{n+1}, \\ \mathbf{h}^{n+1} = -K\nabla \cdot ((\frac{(\phi_2^n)^2}{2}\nabla \mathbf{p}^{n+1}) + \frac{(\phi_2^n)^2}{2}(-h_1 + h_2\|\mathbf{p}^n\|^2)\mathbf{p}^{n+1}, \\ \frac{\partial \mathbf{p}^{n+1}}{\partial \mathbf{n}}|_{\partial\Omega} = 0, \end{array}\right. \tag{7}$$

2. Step 2: update (ϕ_1^{n+1}, ϕ_3^{n+1}):

$$\left\{\begin{array}{l} \frac{\phi_i^{n+1}-\phi_i^n}{\delta t} + \nabla \cdot (\mathbf{v}^n \phi_i^n) = \nabla \cdot (\lambda_i \nabla(\mu_i^{n+1} - \mu_2^n)), \\ \mu_3^{n+1} = C_3(\phi_3^{n+1} - \phi_3^n) + 3\sqrt{2}\gamma_{3s}\varepsilon\left(-\nabla^2\phi_3^{n+1} + f_s'(\phi_3^n)\right) + \gamma_{123}(\phi_1^2\phi_2^2\phi_3)^n, \\ \mu_2^n = 3\sqrt{2}\gamma_{2s}\varepsilon\left(-\nabla^2\phi_2^{n+1} + f_s'(\phi_2^n)\right) + \gamma_{123}(\phi_1^2\phi_2\phi_3^2)^n \\ +\phi_2^n\Big(\frac{K}{2}(\nabla \mathbf{p}^{n+1})^2 + \frac{h_2}{4}\|\mathbf{p}^{n+1}\|^4 - \frac{h_1}{2}\|\mathbf{p}^{n+1}\|^2\Big), \\ \mu_1^{n+1} = C_2(\phi_1^{n+1} - \phi_1^n) + 3\sqrt{2}\gamma_{1s}\varepsilon\left(-\nabla^2\phi_1^{n+1} + f_s'(\phi_1^n)\right) + \gamma_{123}(\phi_1\phi_2^2\phi_3^2)^n + 3\sqrt{2}\gamma_{1b}\Big(\\ \nabla^4\phi_1^{n+1} - f_b'(\phi_1^n)\nabla^2\phi_1^n - \nabla^2 f_b(\phi_1^n) + f_b'(\phi_1^n)f_b(\phi_1^n)\Big) - \alpha_1\nabla \cdot \left((\mathbf{p}^{n+1} \cdot \nabla\phi_1^n)\mathbf{p}^{n+1}\right), \\ \frac{\partial \phi_i^{n+1}}{\partial \mathbf{n}}|_{\partial\Omega} = 0, \frac{\partial \nabla^2\phi_i^{n+1}}{\partial \mathbf{n}}|_{\partial\Omega} = 0, \quad \frac{\partial \mu_i^{n+1}}{\partial \mathbf{n}}|_{\partial\Omega} = 0, \end{array}\right. \tag{8}$$

3. Step 3: update ϕ_2^{n+1}:

$$\phi_2^{n+1} = 1 - \phi_1^{n+1} - \phi_3^{n+1}. \tag{9}$$

4. Step 4: update ($\mathbf{v}^{n+1}$, p^{n+1}):

$$\left\{\begin{array}{l} \rho\frac{\tilde{\mathbf{v}}^{n+1}-\mathbf{v}^n}{\delta t} + (\mathbf{v}^n \cdot \nabla)\tilde{\mathbf{v}}^{n+1} = \eta\nabla \cdot (\tilde{\mathbf{v}}^{n+1} + (\tilde{\mathbf{v}}^{n+1})^T) - \nabla p^n - \phi^n\nabla\mu^{n+1} - \mathbf{h}^{n+1}\nabla \mathbf{p}^n \\ +\nabla \cdot (-\frac{a}{2}(\mathbf{p}^n\mathbf{h}^{n+1} + \mathbf{h}^{n+1}\mathbf{p}^n) + \frac{1}{2}(\mathbf{p}^n\mathbf{h}^{n+1} - \mathbf{h}^{n+1}\mathbf{p}^n) + \zeta\phi_2^{n+1}\phi_3^{n+1}\mathbf{p}^{n+1}\mathbf{p}^{n+1}), \\ \tilde{\mathbf{v}}^{n+1}|_{\partial\Omega} = 0. \end{array}\right. \tag{10}$$

$$\left\{\begin{array}{l} \frac{\mathbf{v}^{n+1}-\tilde{\mathbf{v}}^{n+1}}{\delta t} = -\nabla(p^{n+1} - p^n), \\ \nabla \cdot \mathbf{v}^{n+1} = 0, \quad \mathbf{v}^{n+1}|_{\partial\Omega} = 0. \end{array}\right. \tag{11}$$

In the above, C_2 and C_3 are numerical stabilizing parameters [11]. The above scheme is constructed by combining several effective approaches in the approximation of Cahn-Hilliard equations [11], Navier-Stokes equations [6] and phase-field models [12, 2].

The numerical scheme is further discretized in space by central finite differences, and implemented on graphic processing units (GPUs) using the CUDA interface. The Nvidia CUFFT, Thrust [7], as well as CUSP [1] have been used to solve the linearized systems. The resultant solver is tested in both time and space to ensure it is convergent and attains first order accuracy.

References

[1] N. Bell and M. Garland. Cusp: Generic parallel algorithms for sparse matrix and graph computations. *preprint*, 0:0, 2012.

[2] F. Boyer and C. Lapuerta. Study of a three component Cahn-Hilliard flow model. *ESAIM: Mathematical Modeling and Numerical Analysis*, 40(4):653–687, 2006.

[3] P. G. de Gennes and J. Prost. *The Physics of Liquid Crystals*. Oxford University Press, 1993.

[4] J. Etienne, J. Fouchard, D. Mitrossilis, N. Bufi, P. Durand-Smet, and A. Asnacios. Cells as liquid motors: mechanosensitivity emerges from collecive dynamics of actomyosin cortex. *PNAS*, 112(9):2740–2745, 2015.

[5] W. H. Grover, A. K. Bryan, M. Diez-Silva, S. Suresh, J. M. Higgins, and S. R. Manalis. Measuring single-cell density. *PNAS*, 108(27):10992–10996, 2011.

[6] J. L. Guermond, P. Minev, and J. Shen. An overview of projection methods for incompressible flows. *Comput. Methods Appl. Mech. Engrg.*, 195:6011–6045, 2006.

[7] J. Hoberock and N. Bell. Thrust: a parallel template library. *preprint*, 0:0, 2010.

[8] H. Karcher, J. Lammerding, H. Huang, R. T. Lee, R. D. Kamma, and M. R. Kaazempur-Mofrad. A three dimensional viscoelastic model for cell deformation with experimental verification. *Biophysical Journal*, 85:3336–3349, 2003.

[9] B. Klus, U. A. Laudyn, M. A. Karpierz, and B. Sahraoui. All optical measurement of elastic constants in nematic liquid crystals. *Optics Express*, 22(24):20357–30266, 2014.

[10] G. Salbreux, G. T. Charras, and E. Paluch. Actin cortex mechanics and cellular morphogenesis. *Trends in Cell Biology*, 22(10):536–545, October 2012.

[11] J. Shen and X. Yang. Numerical approximation of Allen-Cahn and Cahn-Hilliard equations. *Discrete and Continuous Dynamical Systems Series B*, 28(4):1669–1691, 2010.

[12] J. Shen and X. Yang. A phase-field model and its numerical approximation for two-phase incompressible flows with different densities and viscositites. *SIAM Journal of Scientific Computing*, 32:1159–1179, 2010.

[13] R. Simson, E. Wallraff, J. Faix, J. Niewohner, G. Gerisch, and E. Sackmann. Membrane bending modulus and adhesion energy of wild-type and mutant cells of dictyostelium lacking talin or cortexilllins. *Biophysical Journal*, 74:514–522, 1998.

[14] W. Strychalski and R. D. Guy. A computational model of bleb formation. *Mathematical Medicine and Biology*, 30:115–130, 2013.

[15] Y. Sun and C. Beckermann. Sharp interface tracking using the phase-field equation. *Journal of Computational Physics*, 220:626–653, 2007.

[16] A. V. Zakharov, M. N. Tsvetkova, and V. G. Korsakov. Elastic properties of liquid crystals. *Physics of the Solid State*, 44(9):1795–1801, 2002.
