# Supplementary material for: Modeling the Excess Cell Surface Stored in a Complex Morphology of Bleb-Like Protrusions
Source: PLoS Comput Biol. 2016 Mar 25;12(3):e1004841. doi: 10.1371/journal.pcbi.1004841 (PMC4807848; doi:10.1371/journal.pcbi.1004841)
Supplement: S1 Fig — Images taken along Z direction from cell bottom with 1um step. (PDF) [file pcbi.1004841.s003.pdf]

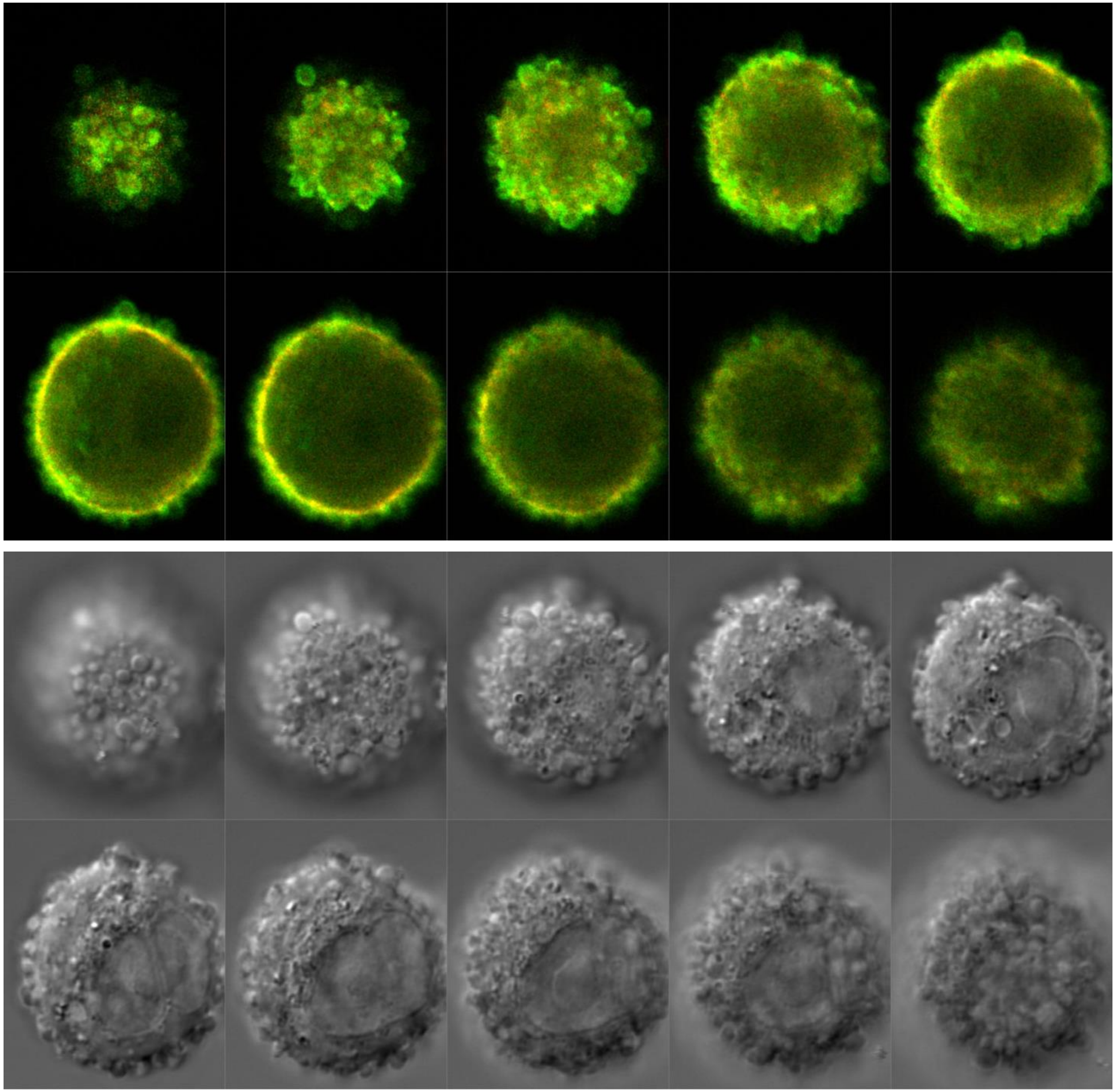

S1 Fig. Fluorescence and DIC imaging of rounded CHO cell stably expressing RFP-Myosin and GFP-Lifeact. Images taken along Z direction from cell bottom with 1um step.
