# Supplementary material for: Modeling the Excess Cell Surface Stored in a Complex Morphology of Bleb-Like Protrusions
Source: PLoS Comput Biol. 2016 Mar 25;12(3):e1004841. doi: 10.1371/journal.pcbi.1004841 (PMC4807848; doi:10.1371/journal.pcbi.1004841)
Supplement: S2 Fig — i.) Initial condition: both the cell surface perimeter, L, and cortex perimeter, Pinitial, start out at identical, coincident positions enclosing area A. N contact points are equally distributed around the perimeter. The inner yellow circle represents the target area, Atarget, to which the cortex will shrink during the computation, imitating a two-dimensional cell rounding. ii) A snapshot at the early stages of computation when inner layer already shrunk to target area with perimeter, Pfinal. Because the inner and outer layers are connected via contact points, folds(BLiPs) are formed around the 2D cell periphery but bending energy of folds is not minimized yet. iii) The final steady state is achieved by minimizing the Hamiltonian (in effect, the curvature of the outer, cell surface layer). In this configuration, contact points on the outer and inner layer meet and, because curvature energy is minimized, the system ceases to further evolve. (PDF) [file pcbi.1004841.s004.pdf]

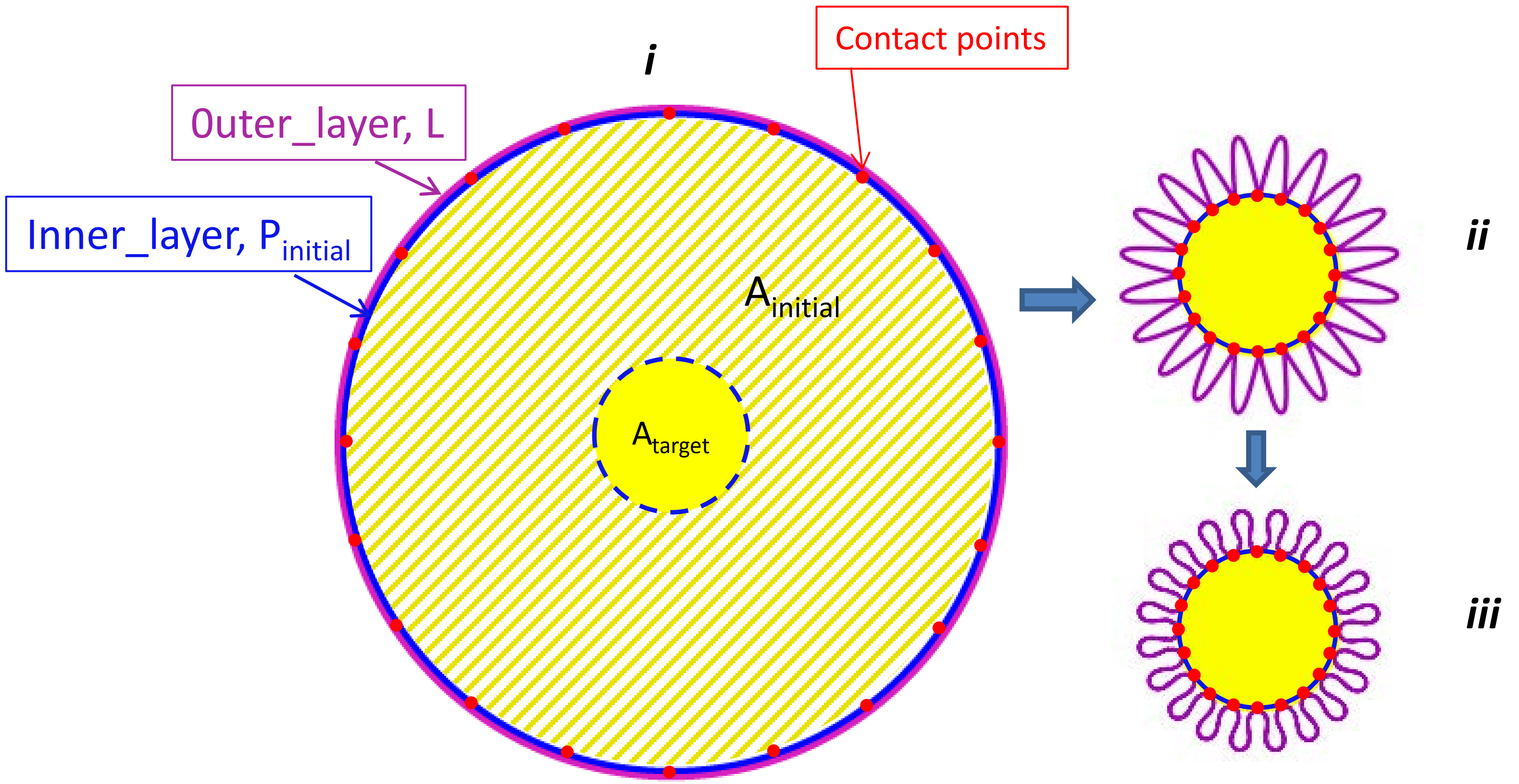

S2 Fig. Schematic of the computational progression from initial (*i*) to final state (*iii)* for the geometric model. *i*.) Initial condition: both the cell surface perimeter, L, and cortex perimeter, $P_{initial}$, start out at identical, coincident positions enclosing area A. N contact points are equally distributed around the perimeter. The inner yellow circle represents the target area, Atarget, to which the cortex will shrink during the computation, imitating a two-dimensional cell rounding. *ii*) A snapshot at the early stages of computation when inner layer already shrunk to target area with perimeter, P_{final}. Because the inner and outer layers are connected via contact points, folds(BLiPs) are formed around the 2D cell periphery but bending energy of folds is not minimized yet. *iii*) The final steady state is achieved by minimizing the Hamiltonian (in effect, the curvature of the outer, cell surface layer). In this configuration, contact points on the outer and inner layer meet and, because curvature energy is minimized, the system ceases to further evolve.
