# Supplementary material for: Modeling the Excess Cell Surface Stored in a Complex Morphology of Bleb-Like Protrusions
Source: PLoS Comput Biol. 2016 Mar 25;12(3):e1004841. doi: 10.1371/journal.pcbi.1004841 (PMC4807848; doi:10.1371/journal.pcbi.1004841)
Supplement: S3 Fig — A. Three overlapping shapes, each has the same area and initial excess ratio ER = 4. The shapes were prescribed to have different number of folds: 20 (yellow), 40 (red), 50 (blue). The blue shape has smaller local radius along the perimeter which results in higher bending energy. B. Three overlapping shapes, each has the same area and number of folds, N = 40. The shapes have different initial excess ratio: 6 (yellow), 4 (red), 2 (blue). The blue shape has smaller local radius along the perimeter which results in higher bending energy. C. Plot of bending energy for simulated shapes with different ERs and number of folds. D. Plot of area stored in BLiPs for different shape configurations. (PDF) [file pcbi.1004841.s005.pdf]

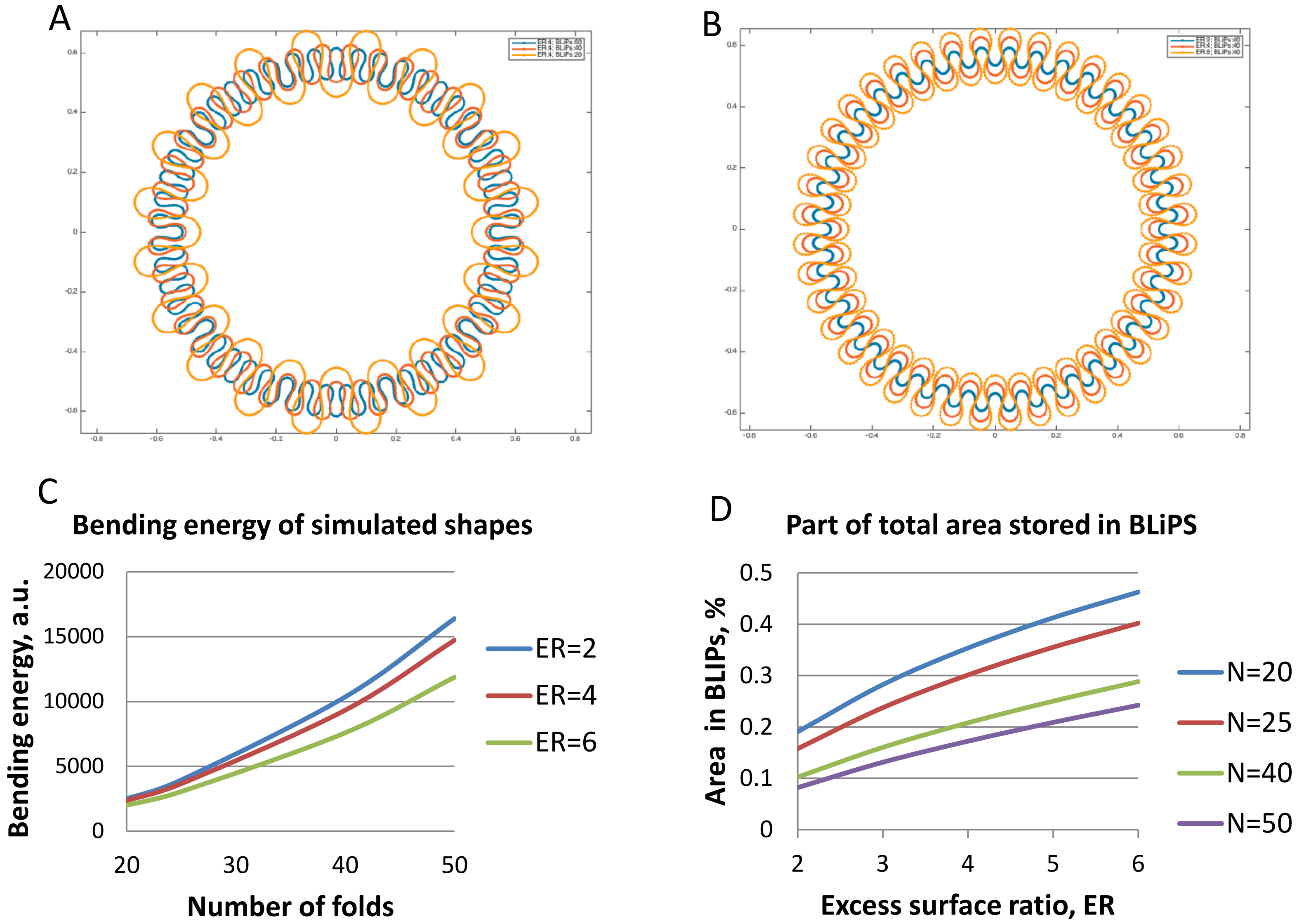

S3 Fig. A. Three overlapping shapes, each has the same area and initial excess ratio ER=4. The shapes were prescribed to have different number of folds: 20 (yellow), 40 (red), 50 (blue). The blue shape has smaller local radius along the perimeter which results in higher bending energy. B. Three overlapping shapes, each has the same area and number of folds, N=40. The shapes have different initial excess ratio: 6 (yellow), 4 (red), 2 (blue). The blue shape has smaller local radius along the perimeter which results in higher bending energy. C. Plot of bending energy for simulated shapes with different ERs and number of folds. D. Plot of area stored in BLiPs for different shape configurations.
