# Supplementary material for: Modeling the Excess Cell Surface Stored in a Complex Morphology of Bleb-Like Protrusions
Source: PLoS Comput Biol. 2016 Mar 25;12(3):e1004841. doi: 10.1371/journal.pcbi.1004841 (PMC4807848; doi:10.1371/journal.pcbi.1004841)
Supplement: S4 Fig — Part of cells was spread on glass bottom dish for 24 hours and another part was detached and plated on the same dish 20min before fixation. Bar = 10. (PDF) [file pcbi.1004841.s006.pdf]

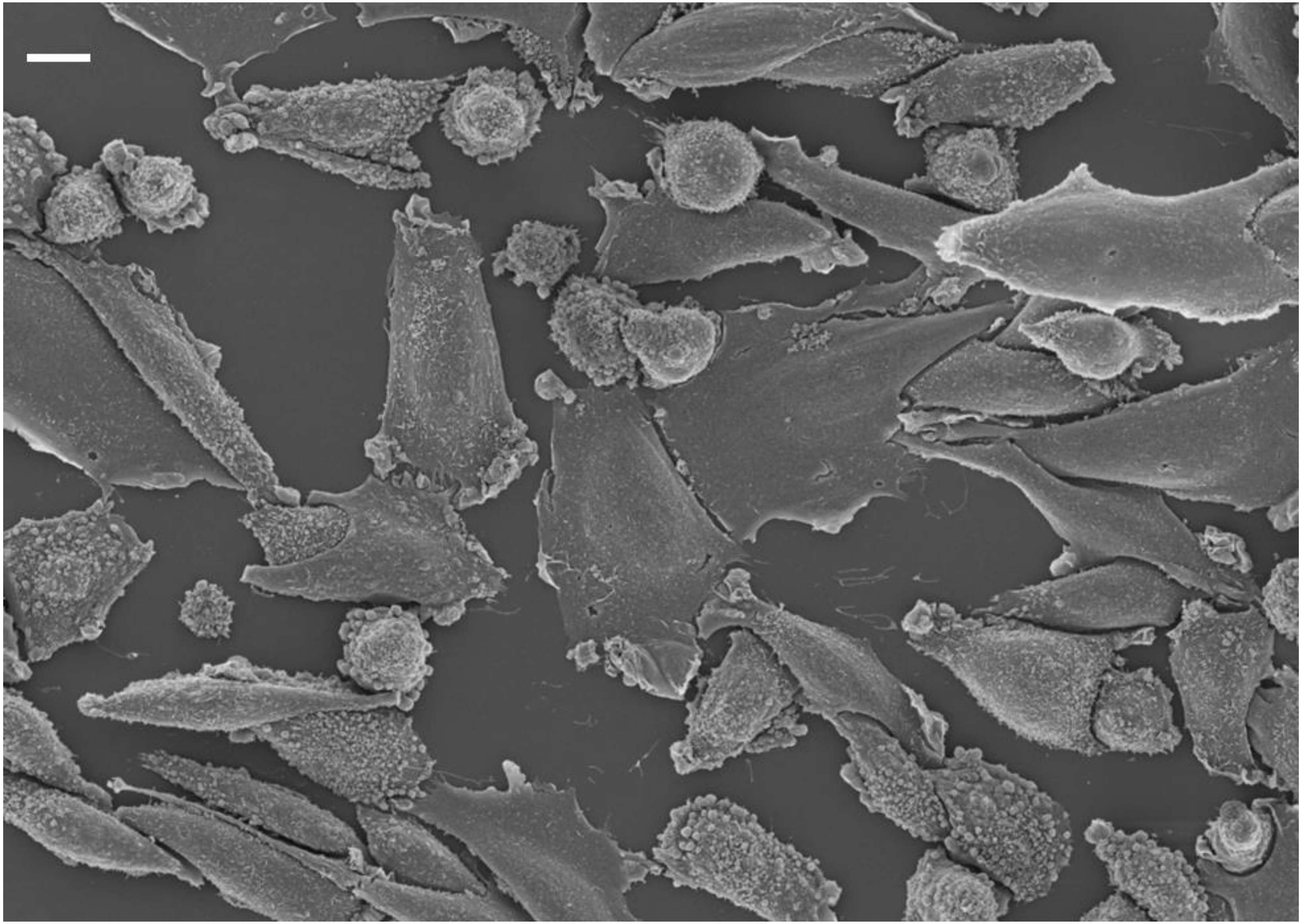

S4 Fig. A SEM image of CHO cells. Part of cells was spread on glass bottom dish for 24 hours and another part was detached and plated on the same dish 20min before fixation. Bar=10 µm
