# Supplementary material for: Modeling the Excess Cell Surface Stored in a Complex Morphology of Bleb-Like Protrusions
Source: PLoS Comput Biol. 2016 Mar 25;12(3):e1004841. doi: 10.1371/journal.pcbi.1004841 (PMC4807848; doi:10.1371/journal.pcbi.1004841)
Supplement: S5 Fig — A. SEM image of rounded CHO cells. B. Enlarged part of image on A (yellow dotted line) with some BLiPs contoured as example of how radius was estimated. Using ImageJ the area within each contour was measured and the radius was calculated assuming that the measured area represents a great circle of BLiP. C. Distribution of BLiPs radii of all cells on image A estimated as it shown on B. (PDF) [file pcbi.1004841.s007.pdf]

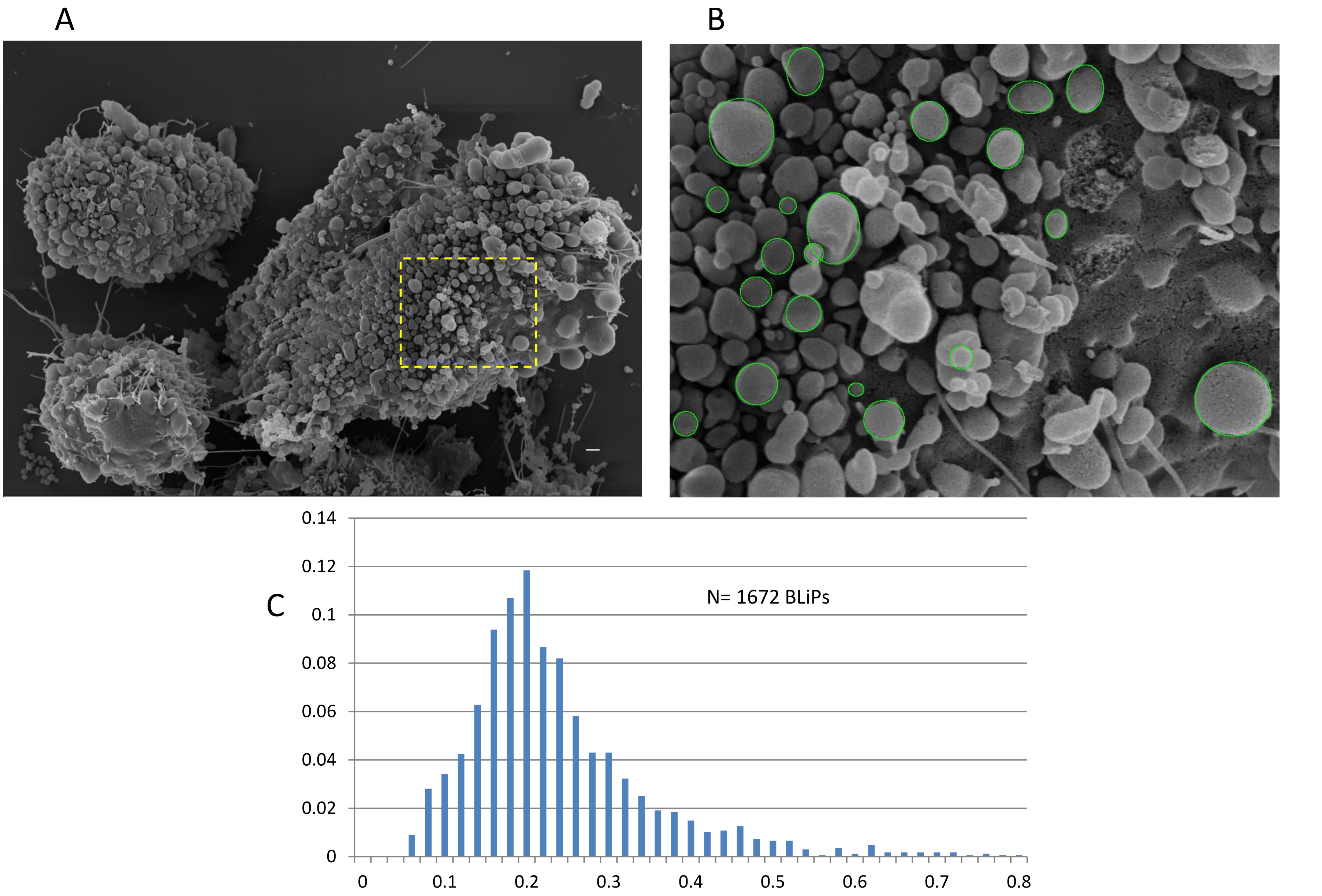


S5 Fig. Estimation of BLiPs sizes. A. SEM image of rounded CHO cells. B. Enlarged part of image on A (yellow dotted line) with some BLiPs contoured as example of how radius was estimated. Using ImageJ the area within each contour was measured and the radius was calculated assuming that the measured area represents a great circle of BLiP. C. Distribution of BLiPs radii of all cells on image A estimated as it shown on B.
