# Supplementary material for: Modeling the Excess Cell Surface Stored in a Complex Morphology of Bleb-Like Protrusions
Source: PLoS Comput Biol. 2016 Mar 25;12(3):e1004841. doi: 10.1371/journal.pcbi.1004841 (PMC4807848; doi:10.1371/journal.pcbi.1004841)
Supplement: S6 Fig — (a) shows the basic cell morphology. The black lines in (b)-(e) denote the direction and magnitude of the nematic director, p where |p| indicates the degree of order between 0 (isotropic) and 1 (maximum order). Here h1/h2 prescribes the Flory order parameter of the nematic equilibrium. Here we choose h1 = 2x10^2^N/m2 and h2 = 2 x 10^2^, 8 x 10^2^, 2 x 10^4^, 2 x 10^6^ N/m2, for (b)-(e) respectively. (PDF) [file pcbi.1004841.s008.pdf]

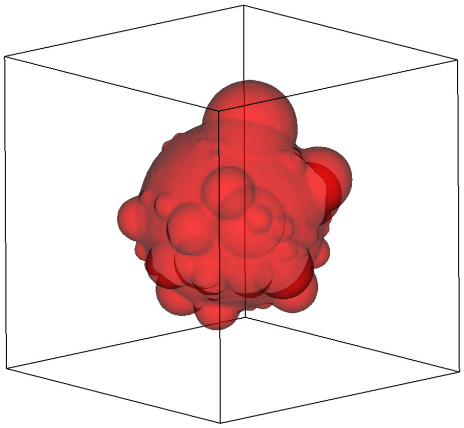

(a) Cell Morphology

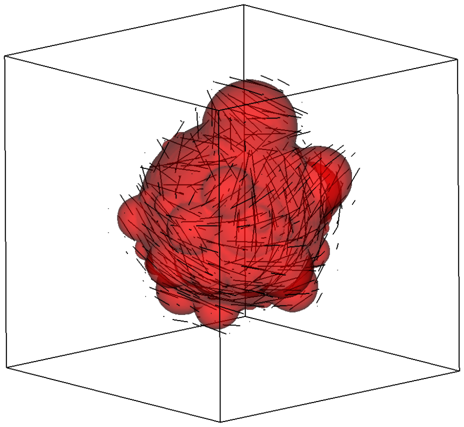

(b) $\sqrt{h_1/h_2} = 1$

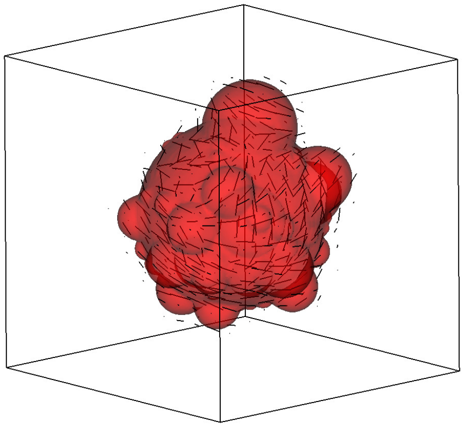

(c) $\sqrt{h_1/h_2} = 0.5$

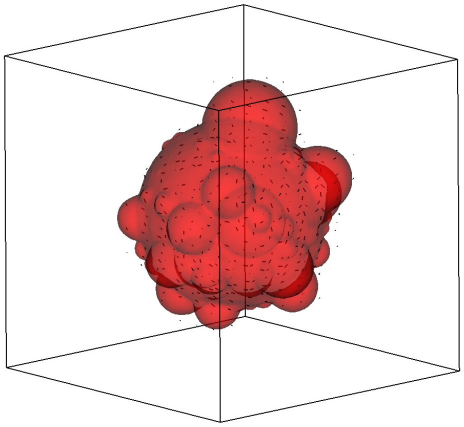

(d) $\sqrt{h_1/h_2} = 0.1$

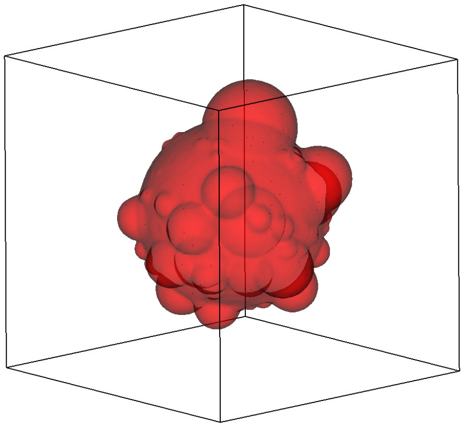

(e) $\sqrt{h_1/h_2} = 0.01$

Figure 6: The black lines in (b)-(e) denote the direction and magnitude of the nematic director, where $|\mathbf{p}|$ indicates the degree of order between 0 (isotropic) and 1 (maximum order). Here $\sqrt{h_1/h_2}$ prescribes the Flory order parameter of the nematic equilibrium. Here we choose $h_1 = 2\times 10^2 N/m^2$ and $h_2 = 2\times 10^2, 8\times 10^2, 2\times 10^4, 2\times 10^6\ N/m^2$, for (b)-(e) respectively.
