# Supplementary material for: Modeling the Excess Cell Surface Stored in a Complex Morphology of Bleb-Like Protrusions
Source: PLoS Comput Biol. 2016 Mar 25;12(3):e1004841. doi: 10.1371/journal.pcbi.1004841 (PMC4807848; doi:10.1371/journal.pcbi.1004841)
Supplement: S7 Fig — (a-c) h1/h2 = 1; (d-f) h1/h2 = 0.5; (g-i) h1/h2 = 0.1. The values of X, Y & Z indicate the position of the 2D slice on the respective axes. (PDF) [file pcbi.1004841.s009.pdf]

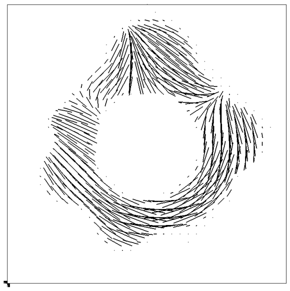

(a) $\sqrt{h_1/h_2} = 1$, $Z = 0.5$

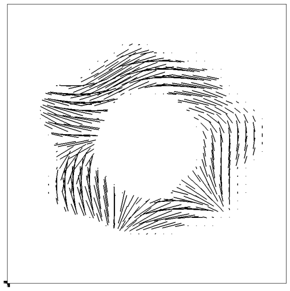

(b) $\sqrt{h_1/h_2} = 1$, $Y = 0.5$

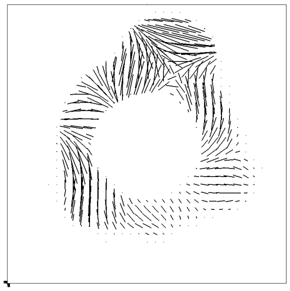

(c) $\sqrt{h_1/h_2} = 1$, $X = 0.5$

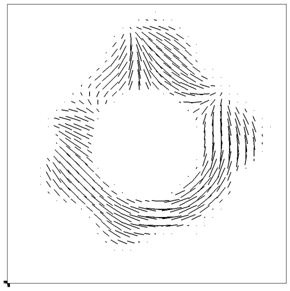

(d) $\sqrt{h_1/h_2} = 0.5$, $Z = 0.5$

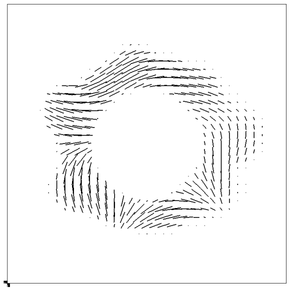

(e) $\sqrt{h_1/h_2} = 0.5$, $Y = 0.5$

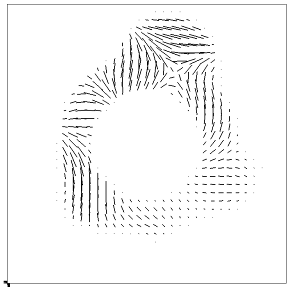

(f) $\sqrt{h_1/h_2} = 0.5$, $X = 0.5$

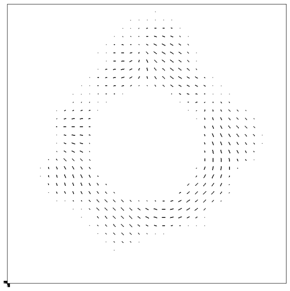

(g) $\sqrt{h_1/h_2} = 0.1$, $Z = 0.5$

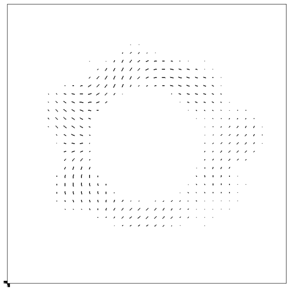

(h) $\sqrt{h_1/h_2} = 0.1$, $Y = 0.5$

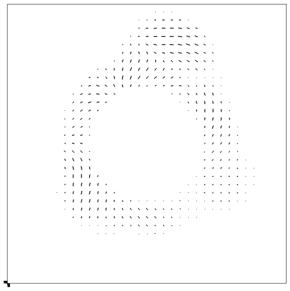

(i) $\sqrt{h_1/h_2} = 0.1$, $X = 0.5$

Figure 7: 2D slices of the surface morphology and director orientation from Figure 1. (a-c) $\sqrt{h_1/h_2} = 1$; (d-f) $\sqrt{h_1/h_2} = 0.5$; (g-i) $\sqrt{h_1/h_2} = 0.1$.
